# Mid-infrared microscopy for label-free digital staining: initial clinical assessment

**DOI:** 10.3389/fbioe.2026.1774279

**Published:** 2026-08-31

**Authors:** L. Duraffourg, H. Borges, M. Fernandes, M. Beurrier-Bousquet, J. Baraillon, B. Taurel, J. Le Galudec, K. Vianey, C. Maisin, L. Samaison, F. Staroz, M. Dupoy

**Affiliations:** 1 Advanced Digital Mid-InfraRed (ADMIR), Moirans, France; 2 Cypath-RB, Villeurbanne, France

**Keywords:** deep learning, digital pathology, digital staining, infrared spectroscopy, microscopy

## Abstract

Infrared (IR) microscopy shows substantial potential for label-free tissue imaging in anatomic pathology, providing rich biochemical contrast. However, existing IR imaging technologies are constrained by slow acquisition speeds and limited spatial resolution. Here, we present a rapid, large-field bimodal imaging platform that integrates conventional brightfield microscopy with a lensless IR imaging scanner, enabling whole-slide IR image stack acquisition in minutes. Using a dedicated deep learning model, we implement an optical H&E staining strategy based on subcellular morpho-spectral fingerprinting. This approach achieves high-resolution visualization of tissue architecture with an effective spatial resolution of 500 nm, without chemical staining. Quantitative metrics, including PSNR (∼24), MS-SSIM (∼0.82), and LPIPS (∼0.22), validate the model's ability to accurately reproduce both the contrast and morphology of cellular structures. Additionally, an initial clinical evaluation on 110 regions of interest within 5 tissue sections demonstrates equivalence between our digital IR-based AI staining and conventional chemical staining, both in image quality and Gleason grading. Together, these results suggest that this IR-based virtual staining approach could provide a fast, chemical-free alternative for anatomic pathology workflows.

## 1 Introduction

Anatomic pathology is the branch of medical science that studies the causes, mechanisms, and effects of diseases. It focuses on observing tissues and cells to understand abnormalities, diagnose diseases, and predict their progression. Pathology serves as a cornerstone of modern medicine, providing insights that guide diagnosis, therapy, and prognosis.

The standard histopathological process flow involves collecting and processing tissue samples through fixation, dehydration, embedding, and thin sectioning, before staining with Hematoxylin and Eosin (H&E). Pathologists then examine the slides under a microscope to identify cellular and structural abnormalities.

The H&E staining is the first step in 95% of cancer diagnoses (Cotran et al., 1999). Complementary tests can be done if necessary. In this case, pathologists take back other sections and redo sample preparations for special staining, which highlights specific components (e.g., Periodic Acid-Schiff (PAS) for carbohydrates, Masson’s Trichrome for collagen …), or for immunohistochemistry (IHC). If necessary, molecular pathology testing is finally performed (Polymerase Chain Reaction – PCR, DNA or RNA sequencing).

Today, the pathologist community is facing three major challenges, namely the ever-increasing demand for tests, the increasing amount of information per test, and the fact that diagnostic error has to be minimized. Moreover, laboratories face a critical shortage of both pathologists and technical personnel. This challenge comes at a pivotal moment, as medicine shifts toward precision and personalized care, an approach that demands a nuanced understanding of cancer’s intricate mechanisms, from the genetic and proteomic levels to the metabolomic landscape (Hackshaw et al., 2020; Senevirathna et al., 2021). For instance, the heterogeneity of cancers has been reported at the expression level of numerous protein markers, at the genetic, epigenetic and transcriptional levels, and more recently at the metabolic and tumor microenvironment levels. Many of these biological characteristics are spatially heterogeneously distributed. Taking account of the different levels of biological heterogeneity (proteomics, molecular, tissue and cellular, metabolic, spatial and temporal) (Gerdes et al., 2014; Kashyap et al., 2022) is essential for improving patient stratification and personalizing treatments.

In this context, the emergence of digital pathology is a natural response to the need for greater efficiency (Hanna and Ardon, 2023) and cancer mechanism understanding. It enables slide image retrieval, report integration, as well as large-scale data mining and research through structured, digitized datasets. Moreover, digital pathology enables the application of artificial intelligence (AI) and quantitative image analysis tools, which may improve diagnostic accuracy, tumor grading, and biomarker quantification. AI-based prognostic models, for example, would offer objective and reproducible assessments that enhance clinical decision-making. Beyond the analysis of known biomarkers, digital pathology holds the potential to accelerate the discovery, validation, and clinical adoption of novel biomarkers, thereby advancing the field of precision medicine.

However, the widespread implementation of digital pathology faces several important challenges and limitations (Sirintrapun, 2025). Some are operational, including the need for high-capacity data storage, substantial financial investment, and the establishment of universal standards and interoperability frameworks. Others are technical, particularly concerning the generalizability and validation of AI algorithms across real-world, heterogeneous datasets. These include variations in scanner hardware, staining protocols across laboratories and the diversity of tissue types. Digitization of complex or special stains also remains technically demanding.

Label-free imaging has recently emerged as a promising way to overcome these technical challenges, thereby accelerating and streamlining the pathology workflow and the integration of AI. In particular, virtual staining of tissue sections that simulates traditional histological staining methods is a popular approach (Bai et al., 2023; Yilmaz et al., 2023; Szulczewski et al., 2024). Once the image is acquired using a slide scanner or a digital microscope, image processing algorithms or artificial intelligence models (Latonen et al., 2024), apply a virtual “stain” that simulates the visual appearance of a conventional chemical staining. Still, virtual staining is facing several significant problems. One relates to the intrinsic variability of AI staining models. Generating accurate nuclei staining remains particularly challenging, as models often either underproduce or overproduce nuclei as it can be noticed in (Koivukoski et al., 2023). Cellular or extra cellular details quality, for instance membrane detail, remains also suboptimal. In addition, the algorithms are constrained to morphological feature analysis and lack the capacity to identify cell types beyond their training data, especially when confronted with abnormal or unseen phenotypes.

To tackle these problems, it has been suggested to replace the traditional brightfield (BF) imaging with alternative microscopy techniques. Approaches such as Quantitative Phase Imaging (QPI)^1^ or ptychography have been suggested as ways to produce more contrasted images (Rivenson et al., 2019; Wang et al., 2024; He et al., 2026). For instance, He et al. propose a hardware-aware programmable Fourier ptychography framework (TAPO), in which the optical acquisition parameters (wavelength, illumination angle, exposure, depth) are jointly optimized with the staining network, reducing acquisition to a small number of task-adapted measurements. However, changing the light source itself could unlock access to a wide range of information.

Indeed, light-tissue interaction, such as absorption and emission, depends intrinsically on the tissue type and cellular elements. These spectral features are intimately related to the macromolecules forming the tissue and can be used to help the AI-models to distinguish different cellular structure. This simple idea has been suggested by several research groups and startups. Among the most promising approaches, autofluorescence imaging (Epi-illumination) seems to be one of the most advanced (Bai et al., 2023). In this case multispectral image stacks corresponding to intrinsic fluorescence of collagen, NADH, flavins and a few others targets are acquired when tissue is irradiated with UV or blue-light. This can be done with standard fluorescent slide scanners with dedicated filters at optical magnification from 20x to 40x, enabling a spatial resolution similar to current BF-scanner resolution (from 500 nm with 20x down to 250 nm typically with 40x). This approach is now the technological baseline of the startup Pictor Labs, which is developing GAN-based AI models for digital staining through style transfer model (Bai et al., 2022). A similar method based on UV fluorescence has also been suggested. The imaging modality uses a simple grayscale reflectance imaging system at low magnification that primarily highlights nuclei (at 365 nm-excitation wavelength). This approach is suggested by startups like MUSE microscopy or Vivascope (Ye et al., 2023; Di Fabrizio et al., 2025). The main applications are in dermatology or at the surgery block for analyzing thick or opaque samples. Such techniques offer lateral spatial resolution that ranges from 250 nm to 1 µm, but suffer from slow acquisition rate. Depending on the modality and magnification, large-area imaging can remain slow; at high magnification, some UV fluorescence/reflectance microscopy approaches report acquisition times on the order of several tens of minutes per cm^2^ (Ye et al., 2023). While more recent systems have since demonstrated higher throughput, simultaneously achieving wide coverage, sub-micron resolution, and biochemical contrast remains a challenge.

Similarly, Raman and Infrared spectroscopies have been explored as approaches for digital staining. Despite being used by the startup Lightcore Technologies for a single test in digital staining (Sarri et al., 2019), Raman spectroscopy remains quite confidential (in terms of publication volume and reported outcomes) for this specific application. On the other hand, IR spectroscopy has extensively been studied as a powerful tool to both perform a digital H&E staining and efficient tissue segmentation (Beć et al., 2020). It has been used since the 1980s in chemistry and physics to analyze materials across the 4,000–400 cm^-1^ range. It is an analytical technique that exploits the interaction of infrared radiation with matter to study and identify chemical compounds. Molecules absorb specific IR frequencies, causing vibrational transitions in their molecular bonds. These absorption patterns are characteristic of a molecule’s structure, making it possible to identify functional groups and determine molecular composition. IR spectroscopy can characterize organic and inorganic compounds in various states (solid, liquid, gas) and is applied in many fields (materials science, pollutant detection, pharmaceutical analysis…).

More recently, IR microscopy has emerged as a versatile analytical tool that combines IR spectroscopy with optical microscopy to enable spatially resolved chemical analysis. It provides detailed insights into the composition and distribution of materials at microscopic scales. Infrared imaging microscopy has then naturally emerged as a valuable tool for the investigation of tissue sections and cell cultures. It provides a direct non-destructive method for chemical identification without the need of additional chemicals (no reagent, no labelling, no staining). The infrared beam passing through a sample reveals the chemical bonds inside proteins, lipids, nucleic acids and more globally primary metabolites (Finlayson et al., 2019; Lorenz-Fonfria, 2020).

Fourier Transform InfraRed (FTIR) microscopes have already demonstrated their ability to image tissues and cells with diagnosis performances that are comparable to conventional approaches such as H&E staining or IHC (Hughes and Baker, 2016). The approach has therefore been validated, including for identifying the grade of certain cancers (Amrania et al., 2018; Ellis et al., 2023).

Such an approach has nevertheless long been limited by the low-power of available polychromatic IR sources. This has made IR microscopes extremely slow for tissue imaging, with acquisition times of several hours for areas of a few mm^2^ (Balan et al., 2019; Finlayson et al., 2019). Moreover, spatial resolution remains at best close to cell size (∼5–10 µm). Recent improvements in uncooled infrared imagers now enable camera-speed acquisitions, without the need for bulky and cryogenic cooling systems (Niklaus et al., 2007). These advancements open new possibilities for spectral imaging in the IR range. With the advent of high-brilliance IR light sources, such as Quantum Cascade Lasers (QCLs) (Faist et al., 1994; Kröger et al., 2014; Vitiello et al., 2015), several optical setups have been suggested and even commercialized this last decade, namely: optical infrared-transmission microscopy, optical photothermal infrared microscopy, and fluorescence-detected Mid-infrared (Mid-IR) photothermal microscopy (Shi et al., 2020). Such approaches have reached spatial resolution around 2 µm (Zhang et al., 2016; Schnell et al., 2020; Xia et al., 2022). Despite compelling demonstrations, these approaches are limited by both low spatial resolution, falling short of pathologists’ requirements for nuclear-level detail and slow acquisition rates (exceeding 10 min per Whole Slide Image, WSI). These two technological limitations currently confine IR microscopy to the proof-of-concept phase, where its use is largely restricted to correlating IR absorption maps with immunohistochemical biomarker analyses. Without a paradigm shift, the path to routine use of IR spectroscopy for cancer segmentation in diagnostic and prognostic workflows may remain long and complex.

This study presents a digital H&E staining method based on a bimodal microscopy approach, coupled with an AI model that leverages both morphological features and IR spectral signatures, the ‘morpho-spectral fingerprint’, to enhance diagnostic precision in anatomic pathology. This method may represent the paradigm shift needed, and our results demonstrate its potential to standardize IR spectroscopy for clinical use. We introduce a novel multimodal imaging framework that integrates an infrared lens-free scanner (Mathieu et al., 2021), with conventional visible-light microscopy. This approach enables rapid wide-field imaging (1 min/1 cm^2^) while leveraging deep learning to generate digitally stained images with a sub-micron (500 nm) effective pixel size.

This paper presents a comprehensive analysis of our approach across three aspects: instrumentation specifications, mathematical evaluation of our deep learning model (metrics), and biostatistical assessment of preclinical prostate tissue data. The results are compared with autofluorescence-based methods, which represent the current state of the art.

## 2 Materials and methods

### 2.1 Biological samples

For this demonstration, we focused our work on prostate cancer. The study included fifteen patients, from which fifteen 3 µm-thick tissue sections were prepared. The unstained formalin-fixed paraffin-embedded (FFPE) tissue sections were deposited on microscopy slides made from Calcium fluoride (CaF_2_). These slides are transparent in the visible and mid-IR ranges. Detailed information about the 15 biological samples are enclosed in Supplementary Material (SI).

Once prepared, unstained slides are imaged in mid-IR light, and in visible brightfield. Once acquisitions are completed, all tissues sections undergoes H&E histochemical staining. These stained slides are once again acquired in BF. Thus, for each tissue section, three images are acquired: unstained infrared, unstained brightfield, and H&E stained brightfield. This last image serves as ground-truth for our deep learning (DL) model.

### 2.2 Image acquisition protocol and data set construction

**FIGURE 1:**
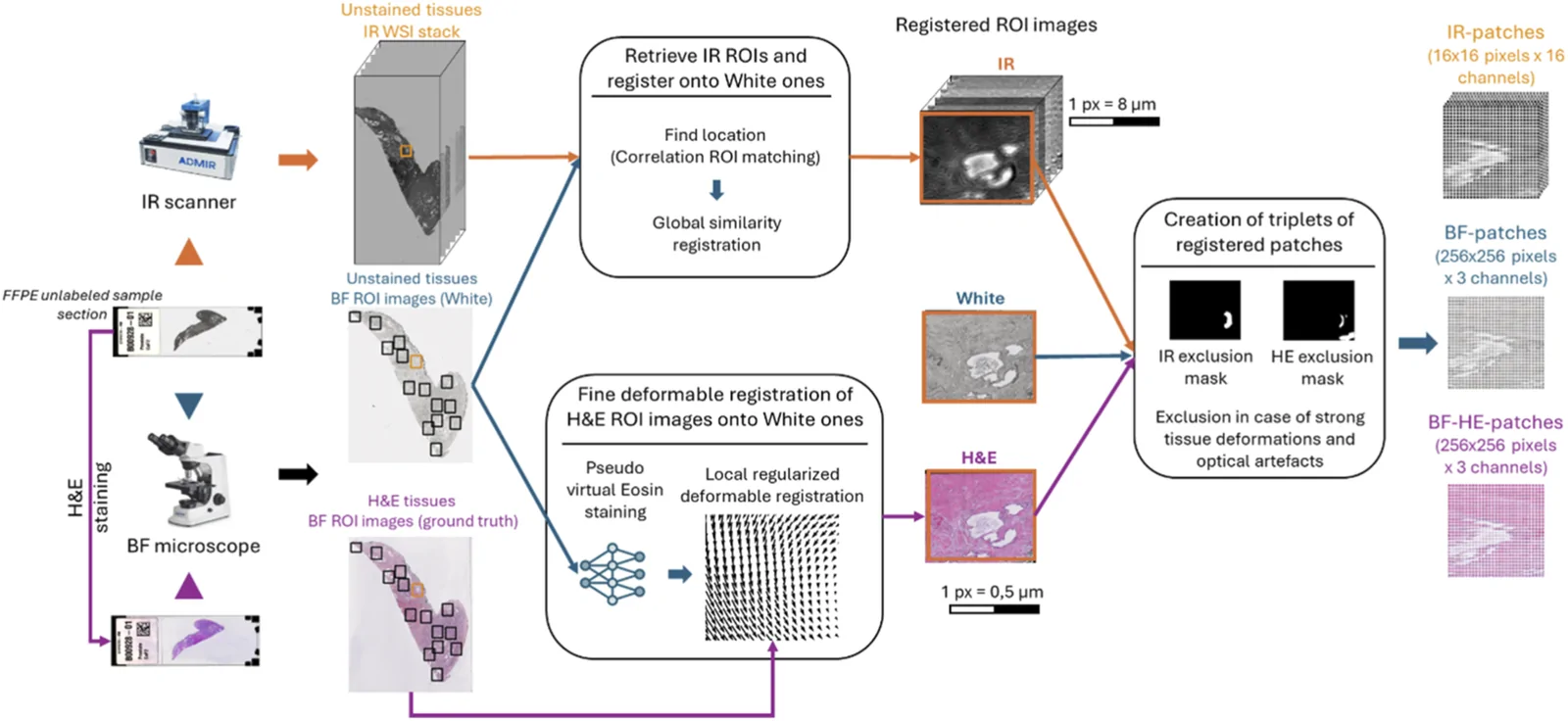
Synopsis of the complete method to prepare the image set for training the DL model – We automatically scan a ROI of a unlabeled tissue section with the BF-microscope. We scan the same tissue section with the IR-scanner at 16 wavelengths to build the IR WSI stack – Once these acquisitions done, this section is H&E stained with a chemical stainer and we scan the same ROIs with the BF-microscope – The BF-ROIs, both pre- and post-H&E staining, are aligned via an automated registration pipeline. This process integrates an eosin-style transfer deep learning model, histogram matching, and rigid-to-deformable image registration – IR-ROIs are extracted from the IR-WSI stack using a custom algorithm that retrieves their positions within the IR WSIs. The resulting IR unstained ROI stacks are then registered to their corresponding BF counterparts. This yields a fully aligned triplet for each ROI. The process is repeated for all ROIs across every tissue section processed by our system.

The overall workflow for image acquisition and processing is illustrated in the Figure 1. The acquisition procedure was as follows: (i) Infrared WSI (IR-WSI) were obtained from unstained tissue sections; (ii) BF images were acquired from multiple regions of interest (BF-ROIs) within the same sections; and (iii) following H&E staining of these sections, the corresponding ROIs were re-imaged under bright-field microscopy (BF-H&E-ROIs). ROI selection was automated and randomized, subject to specific spatial constraints. To prevent metric inflation, each ROI was required to include a small margin of background (non-tissue–less than 10%) area. Furthermore, the selection process was designed to ensure representative sampling across the entire slide.

Registration of unstained BF images to ground truth images is a critical step for minimizing training bias and inference errors (Li et al., 2024; Ma et al., 2026). The issue of training pair misalignment is directly addressed through a dedicated registration pipeline achieving an average error below 0.5 µm (one effective pixel size). The BF-H&E-ROIs are first aligned to the BF-unlabeled ROIs through a custom automated registration pipeline, followed by registration of the IR-ROIs to the same BF-ROIs, achieving near-pixel-level alignment (Figure 1). A registration error has been estimated by computing the distance between registered landmarks in the BF-H&E ROIs and the corresponding original landmarks in the unlabeled BF ROIs. The mean registration error is less than 0.5 µm (equivalent to one effective pixel, for more information, see SI). The automated pipeline incorporates an Eosin style-transfer model to consistently infer Eosin staining, effectively converting BF-ROIs into synthetic H&E representations. This process primarily targets the cytoplasm and extracellular matrix with high stability and reproducibility. The structural contours extracted from this inference facilitate image registration with a high degree of reliability. While the results are of insufficient quality for pathological applications, they still allow image registration through classical computer-vision algorithms (see Section 2.2.3). The IR-ROIs are automatically retrieved from homemade method and algorithms that localize the BF-ROI positions onto the IR-WSI and extract them. Prior to the IR-ROI extraction, the 11 µm-effective pixel size of IR images is rescaled to 8 µm (an integer multiple of the BF pixel size: 0.5 µm) via an interpolation algorithm. Finally, binary exclusion masks were manually annotated to delineate regions containing optical artifacts or severe tissue deformations where registration failed to account for staining-induced distortions. The ROIs were then split into patches for deep learning training; any patches intersecting these exclusion masks were discarded to ensure data quality.

The acquisition protocol was realized on the 15 prostate tissue sections. At the end, we got 118,602 triplets of patch images:

- 16x16 pixel IR-patch images – 16 channels (8 µm-pixel size)
- 256x256 BF-patch images – 3 channels (0.5 µm pixel size)
- and 256x256 BF-H&E-patch images – 3 channels (0.5 µm pixel size)

In the following sections, we detail the optical platforms and the image post-processing for generating the image data sets. In the next sections, ‘spatial resolution’ refers to the effective pixel size rather than the formal diffraction-limited resolution. This definition is the most common one in digital pathology and deep learning community. We emphasis that ‘image stack’ means IR-multispectral cube in our application.

#### 2.2.1 Infrared optical setup

**FIGURE 2:**
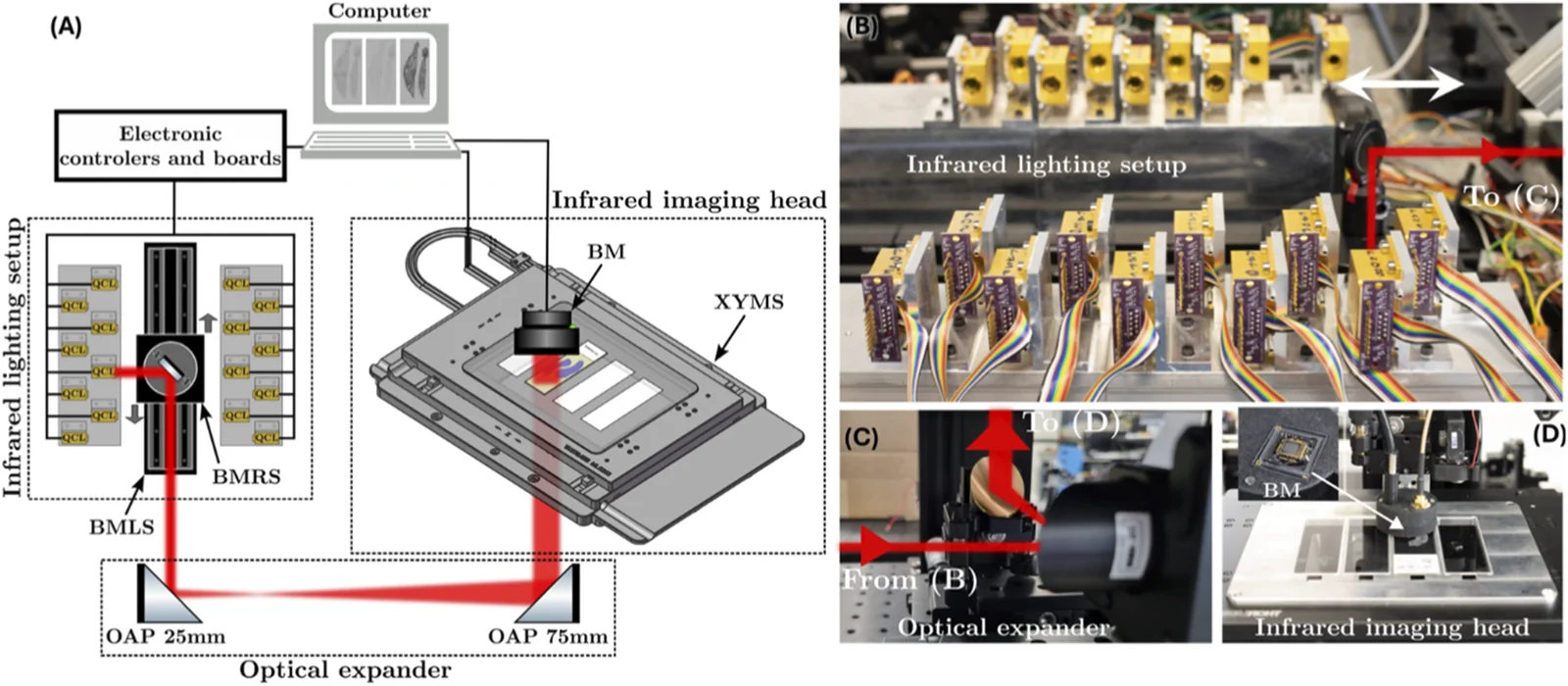
Infrared optical setup: **(A)** Global system including the imaging stage and the lighting setup: The laser beams from QCLs are combined with a flat mirror (Thorlabs - PF10-03-M02) mounted on an automated motorized brushless rotation (BMRS: Thorlabs - DDS300/M) and translation stages (BMLS: Thorlabs - DDR100/M). The beams are expanded using a ×3 afocal optical system consisting of two gold off-axis parabolic (OAP) mirrors with focal lengths of 25 mm and 75 mm to achieve uniform illumination over 3 mm^2^ – optomechanical mounts and control electronics are custom-designed – **(B)** Quantum Cascade laser (HHL packaging) sets forming the illumination stage – **(C)** Beam expander for a homogenous lighting (MPD119-M03 in the foreground) and periscope mirror (in the background MPD139-M03) – **(D)** Sample holder placed on a the XY stage (XYMS: Thorlabs MLS203-1) and IR bolometer matrix (BM) – optomechanical mounts and control electronics are custom-designed.

**FIGURE 3:**
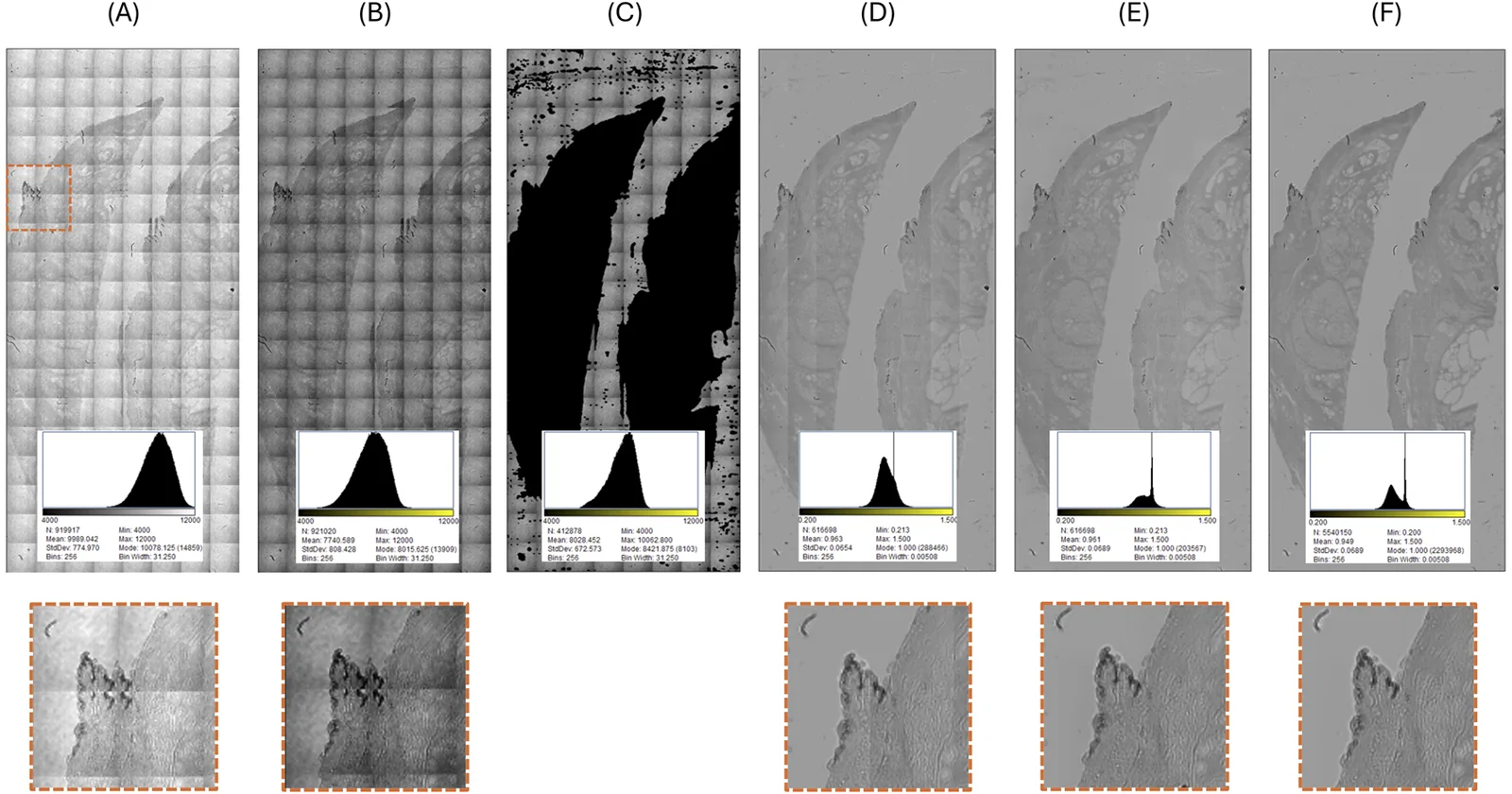
IR-WSI post-processing: **(A)** Raw image built on successive tiles corresponding to the IR camera field of view $I_{raw}$ – **(B)** Offset correction applied on each tile of the image $I_{offset}$ – **(C)** Mask definition to remove dusts and artifacts $I_{background}$ – **(D)** Resulting transmission WSI from Equation 1 $T_{LR}$ – **(E)** Gradient leveling using overlaps to erase the tiling effect $T_{GL}$ – **(F)** Oversampling using subpixel shift acquisition to increase the spatial resolution and get 11 µm-equivalent pixel size $T_{HL}$ .

We have developed a custom-built, multi-wavelength mid-IR illumination system, integrated with a wide-field, lensless imaging setup. The IR platform has been built upon two main parts, Figure 2A:

- The IR source, Figure 2B – It incorporates sixteen discrete QCLs (Faist et al., 1994; Bird and Baker, 2015; Spott et al., 2016; Coutard et al., 2020), each set to emit at wavelengths corresponding to the rovibrational transitions of chemical bonds present in essential biomolecular classes, including nucleic acids (DNA, RNA), proteins, and carbohydrates (Zhizhina and Oieinik, 1972; Barth, 2007; Baker et al., 2014). The laser beams are combined with a flat mirror mounted on an automated motorized brushless rotation and translation stages—optomechanical mounts and control electronics were custom-designed, and are expanded using a 3× afocal optical system—consisting of two gold off-axis parabolic mirrors with focal lengths of 25 mm and 75 mm—to achieve uniform illumination over a 3 mm^2^ area, see Figure 2C. The illumination uniformity is characterized by a contrast $C=\sigma_{I}/I\overset{®}{\;}$ of less than 5%, where $\sigma_{I}$ and $I\overset{®}{\;}$ represent the standard deviation and mean intensity across the illuminated field, respectively. Each QCL is individually controlled by custom-designed electronic boards that provide thermal regulation via a proportional-integral-derivative (PID) feedback loop and sequential activation to image the sample at chosen wavelengths, delivering an average optical power between 20 and 30 mW.
- The IR image acquisition setup–The IR transmission images are realized using an uncooled bolometer array ( 80×80 pixels, 34 µm pitch, NETD 50 mK) with a dedicated packaging (Yon et al., 2014) and a motorized microscopy XY-stage (MLS203 Thorlabs), Figure 2D, providing an image at each wavelength, in grey levels. Targeting a broad spectral range of 5–10 µm (2000–2000 cm^-1^) requires a broadband^2^ camera. However, standard window-equipped sensors are incompatible with our lensless approach, which requires a close sample-to-sensor working distance (typically 300 µm) to prevent image blurring. To overcome this millimeter-scale gap, we employed a camera featuring pixel-level packaging technology, allowing the optically redundant final window to be removed. This lensless transmission architecture has the advantage of eliminating the wavelength-dependent focal plane shifts inherent to refractive-objective systems: the sample-to-sensor distance is fixed and common to all wavelengths. The only residual spectral dependence arises from Fresnel diffraction over this gap, whose effects are characterized in Section 2.1.
- Through the technical features, the infrared camera can be placed close to the sample at <0.5 mm distance. The frame rate is 30 Hz. WSIs are reconstructed from individual image tiles, each corresponding to the camera’s field of view (FOV), with a 5%–20% overlap between adjacent tiles, in both directions.
- A super resolution by sub-pixel shifting approach is used to reach 11 µm effective pixel size (Cheol Park et al., 2003). The spatial resolution is directly set by the camera pitch and the acquisition method as the system does not include any objective. At the end of the acquisition, we get “tiled” WSI as shown in Figure 3A.

The total acquisition time across the spectral range is governed by several parameters: the FOV, which is intrinsically linked to the infrared sensor dimensions, the mechanical scan speed, the frame rate, and the stabilization latencies associated with the QCLs and supporting electronics.

To illustrate the system’s performance without exhaustive derivation, we present two representative tested configurations:

- Configuration 1 (paper’s bolometer array): Utilizing the bolometer (operating at 30 Hz with a 5% frame overlap), the acquisition time was approximately 180 s/cm^2^ for 16 discrete wavelengths. This performance level is sufficient for our proof-of-concept demonstration.
- Configuration 2 (Advanced Focal Plane Array): By employing a secondary focal plane array ( 640×480 pixels, 17 µm pitch, NETD 30 mK) at 60 Hz, the acquisition time was reduced to 1.2 s/cm^2^ for the same spectral density. In this case, the super-resolution step was bypassed, thereby reducing the acquisition overhead.

Following acquisition, a dedicated post-processing pipeline, see Figure 3, was applied to the raw infrared images at each wavelength ( $I_{raw\_\lambda i}$ ) to generate bias-corrected transmission images ( $T_{\lambda i}$ ). The Equation 1 summarizes the correction:

$$T_{\lambda i}=\frac{I_{raw\_\lambda i}-I_{offset}}{I_{bkg\_\lambda i}-I_{offset}}$$ (1)

The offset image $I_{offset}$ is obtained by averaging 5 to 15 successive frames, in absence of illumination (see the correction effect in Figure 3B), while the background image $I_{bkg\_\lambda i}$ at the wavelength $\lambda_{i}$ is computed by averaging several IR image regions that do not contain tissue.

To accurately isolate background areas, we developed an automated segmentation algorithm that excludes both the tissue sample and slide artifacts (dust, defaults …), see the correction effect in Figure 3C. The resulting transmission image $T_{LR}$ is shown in Figure 3D. To smooth the tiling effect, a gradient leveling algorithm (Tweel et al., 2024), that uses overlap areas between adjacent tiles, was applied on $T_{LR}$ to achieve a proper low resolution transmission image $T_{GL}$ , see Figure 3E. This procedure was applied on each sub-pixel shifted images. These were finally averaged on a higher resolved grid to build the high resolution transmission WSI $T_{HR}$ (11 µm-effective pixel size). For each IR-WSI, an histogram is superimposed (see Figures 3A–F). The histograms, which represent the pixel distribution according to the grey level, display clearly two pixel classes corresponding to the background and the tissue. The signal to background ratio is notably enhanced during the whole process. In addition the spatial details are clearly better contrasted after oversampling, as shown in the different zoomed areas below the WSIs. Notice that the spectral consistency of the acquisitions is ensured by two complementary mechanisms: a loop thermal regulation specific to each QCL, which stabilizes source emission, and the correction applied to each wavelength, Equation 1, which compensates for residual source and detector fluctuations as well as slow drifts.

Finally, we get a stack of 16 oversampled IR-WSIs, corresponding to the 16 IR-wavelength used to construct the IR images (see Figure 1) for the training and test of our digital staining model. Spatial sampling of the IR images was enhanced using a two-stage approach:

- Optical Over-sampling: Achieved through sub-pixel displacements of the sample stage, resulting in an effective pixel size of 11 µm in the object plane.
- Numerical Interpolation: To simplify deep learning training, images were then upscaled by interpolation to reach an 8 µm-pixel size (see Figure 1).

#### 2.2.2 Bright field optical setup

The BF image acquisitions were performed with a commercial Zeiss microscope (Axioscope 5) with a 20× $NA_{obj}$ 0.45 objective and a CMOS camera (3.45 µm pitch, 2464×2056 pixels) with an effective pixel size of 274 nm in the object-space. The condenser numerical aperture $NA_{cond}$ was adjusted to match that of the objective.

The microscope has been equipped with a two-axis motorized microscopy stage (MLS203, Thorlabs) to enable automated scanning of tissue sections within the camera’s FOV (300 µm × 300 µm), following an S-pattern trajectory with 5% up to 20% overlap between adjacent fields, thereby generating a composite image of the ROI with a surface of 8377×6990 pixels , i.e. 2.29 mm × 1.91 mm (with 0.5 µm pixel size, corresponding to ×20 magnification). The ROIs were rescaled at ( 4580×9820 pixels ). The registration process slightly adjusted the (x, y) coordinate grids to ensure precise spatial alignment of ROIs (next section).

**FIGURE 4:**
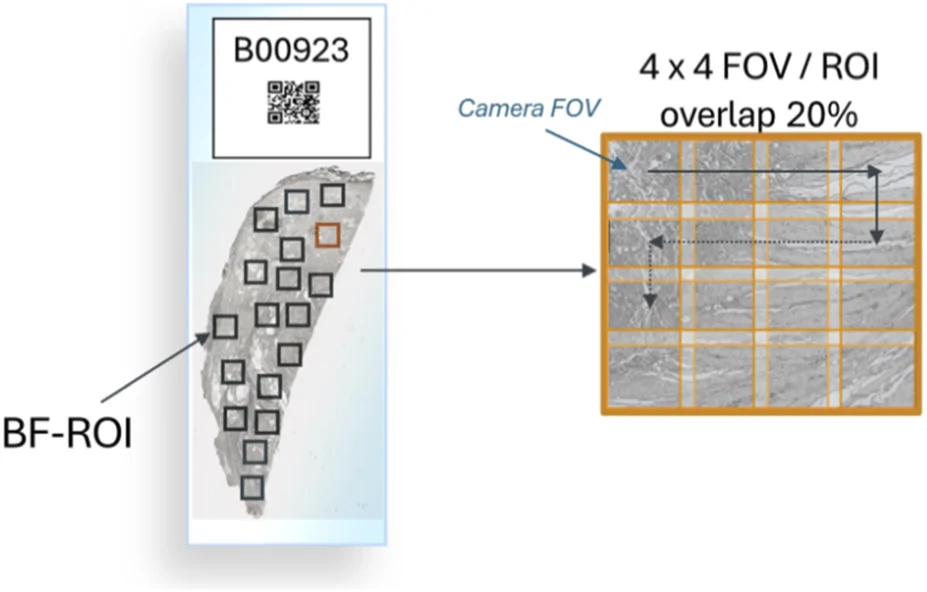
Acquisition method of ROIs from prostate tissue sections.

The acquisition procedure is illustrated in Figure 4.

#### 2.2.3 Image registration

First, BF-ROI and BF-H&E-ROI were registered at a resolution of 0.5 µm per pixel. This step aimed to correct morphological distortions in the native tissue section caused by the histochemical staining process—including hydration, staining, and dehydration.

**FIGURE 5:**
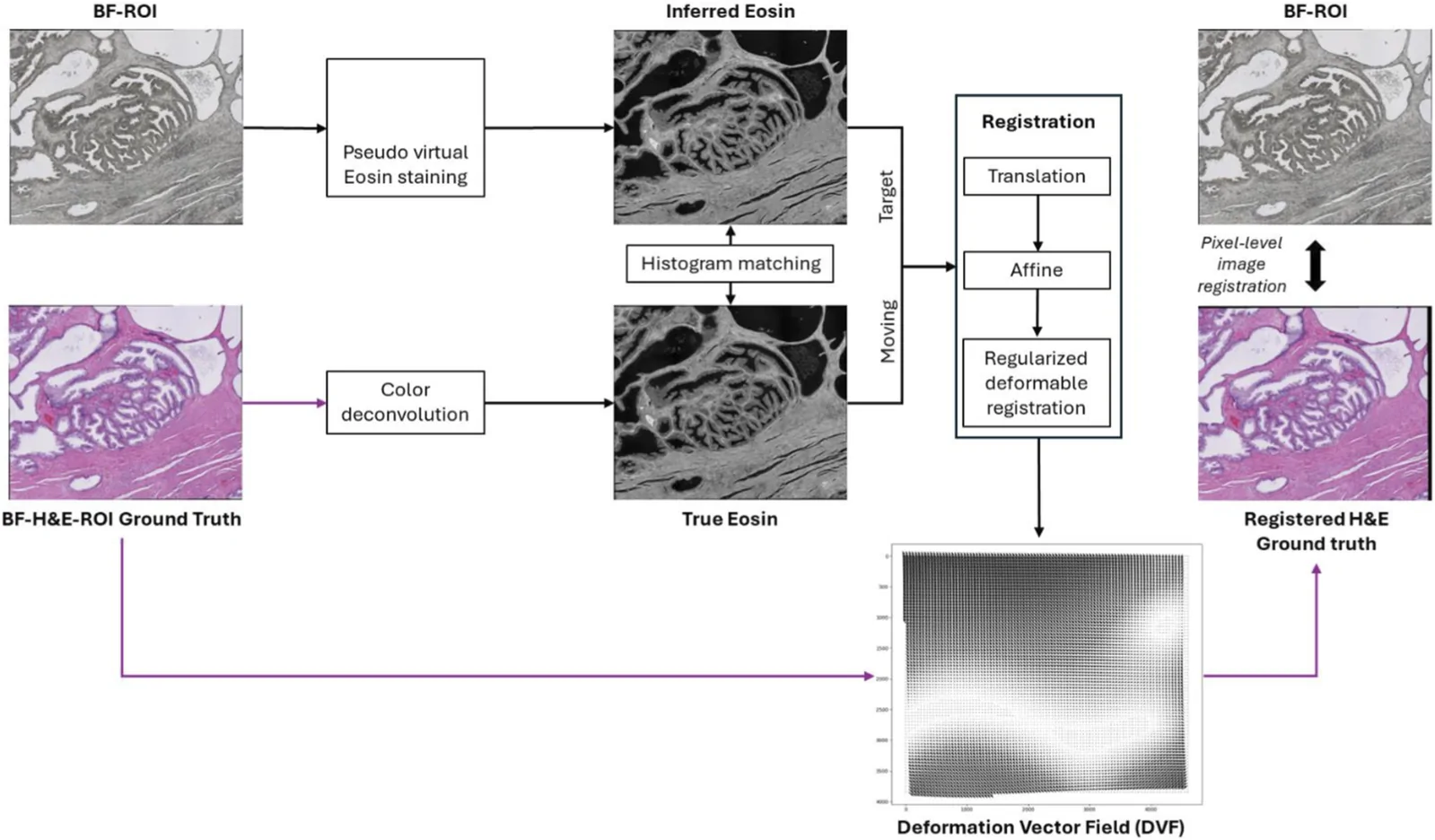
Registration operations between a BF-ROI and the Ground truth BF H&E ROI – The eosin-style transfer model, when applied to brightfield ROIs (BF-ROIs), preserves cytoplasmic eosin information that faithfully represents tissue contours, ensuring fine registration between the inferred eosin ROI and the true eosin ROI.

Manual registration was first conducted by applying a thin-plate spline transformation to the BF-H&E-ROI, calculated from four anatomical landmarks (nucleoli or cell membranes which typical size <0.5 µm, which provided high degree of spatial reliability during the alignment) as reference points. The roughly aligned ROIs were subsequently used to train an automated finer registration pipeline. This pipeline utilized an eosin-style transfer model, including a color (or stain) deconvolution (Ruifrok and Johnston, 2001), and followed by a histogram matching and a sequential rigid-to-deformable registration (translation, affine and B-spline deformable transformations) of the true eosin ROI onto the one inferred from the BF-ROI, as shown in Figure 5. We emphasize that, at this stage of the image processing pipeline, the eosin-style transfer model does not achieve the image quality required for pathologist assessment. This style transfer primarily highlights the cytoplasm, fibers, and the extracellular matrix. While it provides a general structural contour, it lacks the necessary morphological detail and nuclear definition required for definitive diagnostic use.

The second registration consisted of extracting the IR-ROI in the IR-WSI at 8 µm-precision (IR image pixel size) aligned with the BF-ROI. To achieve this, a BF-WSI of the unlabeled tissue section acquired at magnification 5 × served as the cross-reference between the BF-ROI and the IR-ROI. The IR-WSI was first registered onto the BF-WSI rescaled at pixel size 8 µm through an affine transformation calculated from 3 landmarks manually selected. Additionally, the BF-ROI downscaled at magnification ×5 was localized (correlation ROI matching) and finely aligned (similarity transformation) into the original BF-WSI. Based on these findings, the IR-ROI was easily retrieved in the IR-WSI and aligned to the BF-ROI using the inverse (similarity) transformation.

It should be noted that IR-BF registration is performed at IR pixel resolution (8 µm) and is not intended to achieve sub-cellular precision: each IR pixel provides a spectral signature averaged over one to two cells, while fine morphology is carried by the bright-field channel. This division of roles makes the inter-modal registration robust despite the differences in resolution and optical characteristics between the two channels.

### 2.3 Deep learning algorithm architectures

**FIGURE 6:**
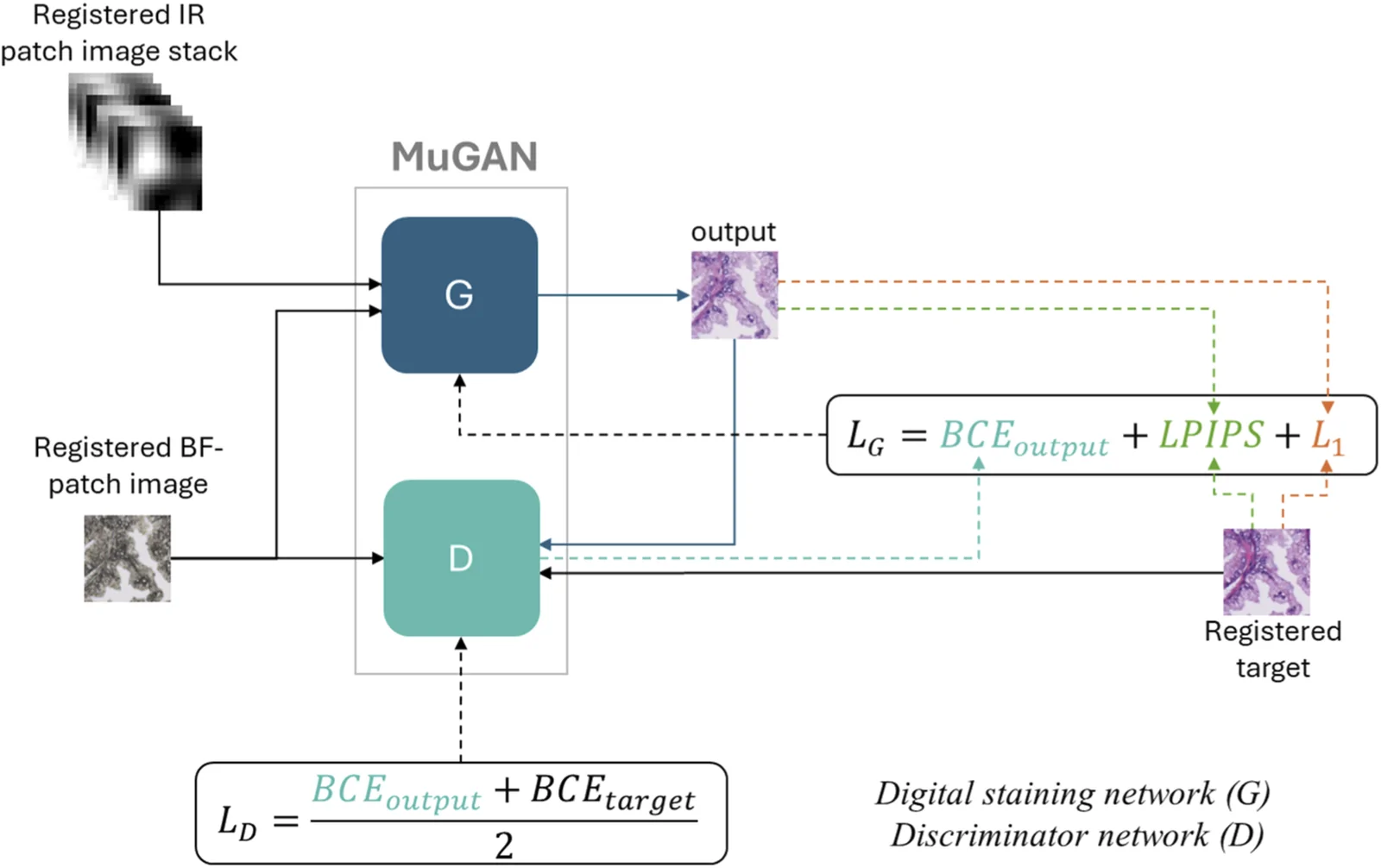
Conditional MUGAN framework composed of an AttMUNet generator (G) and a PatchGAN discriminator (D) with corresponding losses (LG and LD respectively). The IR patches are used only by the generator. BF patches are used by the generator for the inference task. The BF patches are also used as input to the discriminator for the real/fake classification task in order to condition the global model to generate images following the same scene and structure as the input.

**FIGURE 7:**
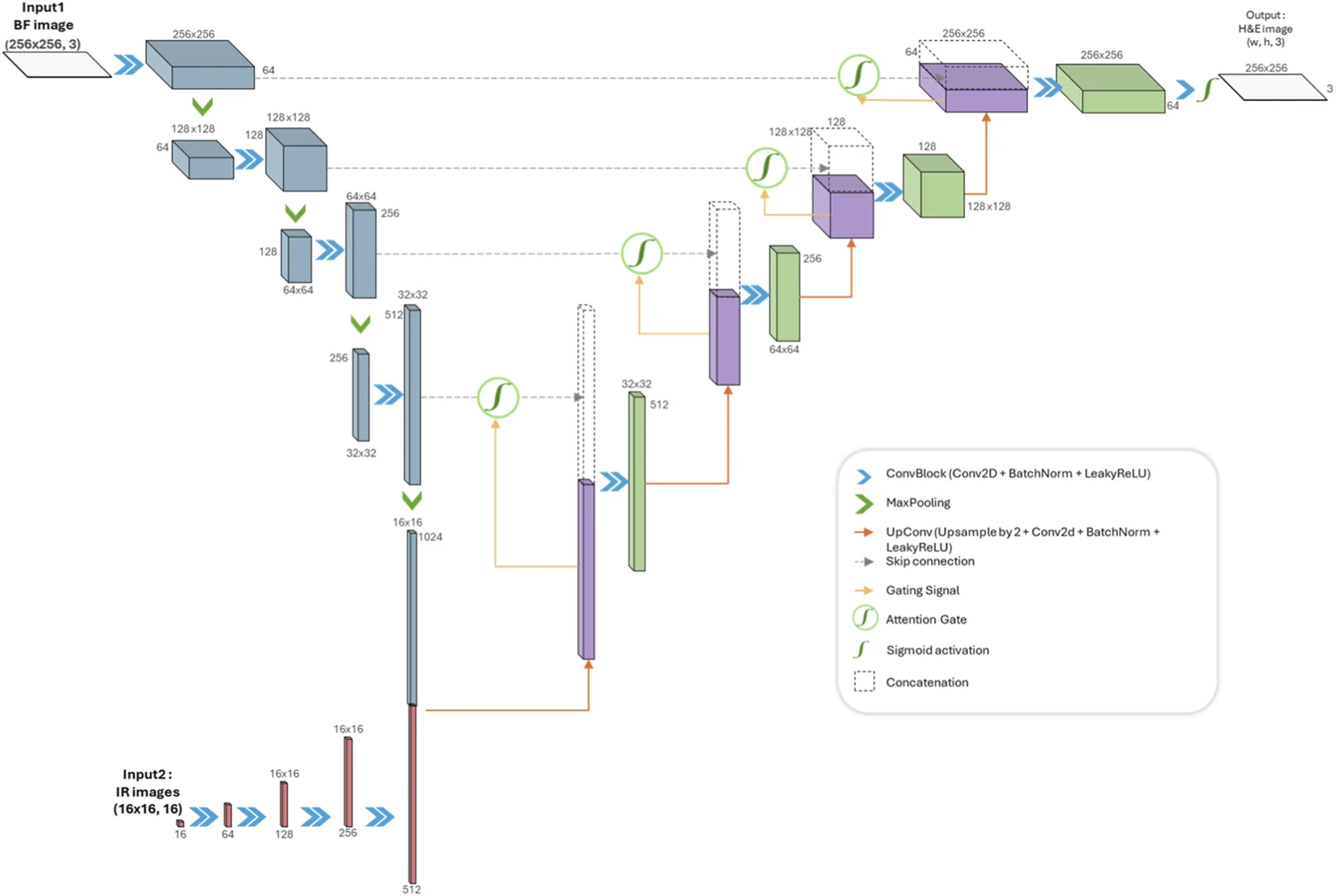
Schematic of the generator (G) based on the AttMUNet.

**FIGURE 8:**
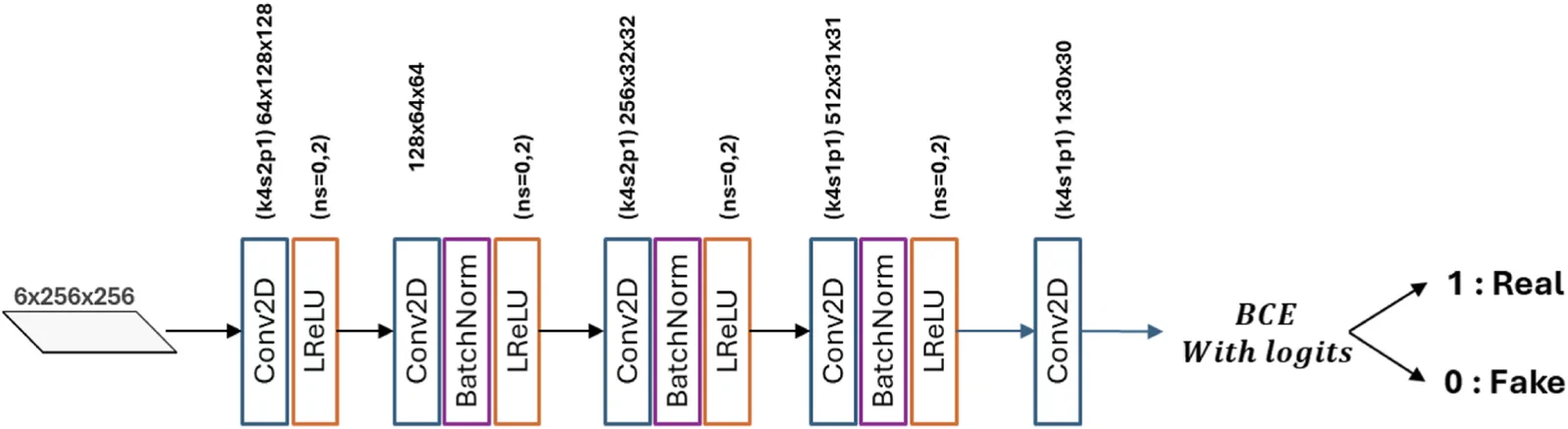
Schematic discriminator (D) PatchGAN – BCE: Binary Cross-Entropy – BCE=1 ↔real image , BCE=0↔Fake image – LRelu: LeakyReLU; Conv2D: 2D convolutional layer.

Our digital pathology model is based on a conditional GAN framework (Mirza and Osindero, 2014). Our generator ( G ) is based on an attention U-Net architecture (Oktay et al., 2018) that we modified for multimodal and multispectral input data and named Attention Multimodal UNet (AttMUNet). We refer to the overall architecture as “MUGAN”. Our discriminator ( D ) is a patchGAN classifier (Isola et al., 2017), see Figure 6. PatchGAN works by splitting a patch into sub-patches. Then, it tries to classify each of these thumbnails as real or fake (in our case: a ground truth chemical H&E, or a digitally stained one). All of these responses are averaged to provide a single response (real or fake) for the entire patch at the output of D . Both generator and discriminator use modules of the form Convolution-BatchNorm-LeakyReLu (see Figures 7, 8).

The AttMUNet (Figure 7) is designed to process and integrate features from both BF image patches and IR image patch stacks. It extracts and fuses morphological and spectral features from these modalities to enable digital H&E staining. Because the BF and IR data have different spatial resolutions (0.5 µm and 8 μm, respectively), the IR data is injected later in the network than the BF data. In other words, they undergo a different number of Conv-BN-LR blocks and are only concatenated once their respective feature maps reach matching dimensions. This ensures that their feature maps have compatible dimensions after the convolution and max-pooling stages, allowing them to be concatenated and passed together to the decoder. The learning rate for optimizing G was set at $1.10^{-4}$ , while for D , it was set at $1.10^{-5}$ . The Adam optimizer was employed for network training, and the batch size was set at 24.

The generator G is trained to produce images that the discriminator D , which is simultaneously trained to distinguish real images from the generator’s fakes, cannot tell apart from real ones. To force the generator to produce an output with the same structure as the BF-patch, both BF and H&E-patch are concatenated before being fed to D . It conditions D based on the BF-patch. We define $x_{BF}$ as BF-patch input, $x_{IR}$ as IR-patch input, x as the pair of inputs $\left(x_{BF},x_{IR}\right)$ , y as the H&E-patch reference and $\tilde{y}$ as H&E output generated by G . The objective of our conditional GAN can be addressed as:

$${L_{cGAN}\left(G,D\right)=E}_{x,y}\left(\log D\left(x_{BF},y\right)\right)+E_{x,y}\left(\log\left(1-D\left(x_{BF},\tilde{y}\right)\right)\right)$$ (2)

In Equation 2, G tries to minimize this objective, and D tries to maximize it, meaning that our global objective $G^{*}=\arg\min_{G}\max_{D}L_{cGAN}\left(G,D\right)$ . We also add a $L_{1}$ (Mean Absolute Error) loss ( $L_{L1}$ ), controlled by a regularization parameter λ (set to 100), to capture low frequency features and a LPIPS (Learned Perceptual Image Patch Similarity) perceptual loss ( $L_{LPIPS}$ ) (Zhang et al., 2018) to capture high frequency features and produce realistic H&E patches. LPIPS acts as a perceptual regularizer, guiding the generator toward a visually realistic optimum that preserves the structural and semantic fidelity essential for reliable digital H&E staining. Our final global objective is now:

$$G^{*}=\arg\min_{G}\max_{D}L_{cGAN}\left(G,D\right)+\lambda L_{L1}\left(G\left(x\right),y\right)+L_{LPIPS}\left(G\left(x\right),y\right)$$ (3)

In Equation 3, $L_{1}$ is defined in the Equation 4:

$$L_{1}=\frac{1}{N}\sum_{i=1}^{N}\left|y_{i}-\tilde{y_{l}}\right|$$ (4)

With N , $y_{i}$ and $\tilde{y_{i}}$ the total number of pixels, the ground truth pixel value at position i and the predicted pixel value at the same position i , respectively.

The *LPIPS* loss is defined in Equation 5 as a distance between two images $A_{1}$ and $A_{2}$ with a network (in this case a Visual Geometry Group neural network–VGG). A stack of L layers is extracted and unit-normalized (designated as ${\hat{f}}_{l}$ ) in the channel dimension for each layer l . The activations are scaled channel-wise by vector $w_{l}$ and the $L_{2}$ distance is then computed:

$$LPIPS\left(A_{1},A_{2}\right)=\sum_{l}\frac{1}{H_{l}W_{l}}\sum_{h=1,w=1}^{H_{l}W_{l}}{\left\|w_{l}⊙\left({\hat{f}}_{l}{\left(A_{1}\right)}_{hw}-{\hat{f}}_{l}{\left(A_{2}\right)}_{hw}\right)\right\|}_{2}^{2}$$ (5)

More specifically, the loss used for training the discriminator (D) was defined as:

$$L_{D}=\frac{BCE\left(D\left(x_{BF},y\right)\right)+BCE\left(D\left(G\left(x\right),y\right)\right)}{2}$$ (6)

In Equation 6, BCE is the Binary Cross Entropy (Mirza and Osindero, 2014) defined for N observations, see the Equation 7 below:

$$BCE=-\frac{1}{N}\sum_{i=1}^{N}\left[z_{i}\log\left({\hat{z}}_{i}\right)+\left(1-z_{i}\right)\log\left(1-{\hat{z}}_{i}\right)\right]$$ (7)

Where $z_{i}\in \left\{0,1\right\}$ is the actual binary label of the $i^{th}$ observation, and ${\hat{z}}_{i}\in \left(0,1\right)$ is the predicted probability that z=1 .

The discriminator should no longer distinguish the real images from the inferred ones during training, and the loss $L_{D}$ theoretically tends to ½.

ROIs from 15 prostate tissue sections were considered. The model was trained with 10 sections from 10 patients and evaluated on 5 sections from different patients. Although inference is performed at the patch scale, with full regions of interest (ROIs) subsequently reconstructed from the predicted patches, the training and testing patches remain biologically and physically independent. This patient-level separation strictly prevents data leakage between the training and testing phases. In total:

- 131,672 triplets of patch images were extracted from 276 ROIs (18.4 ROIS per slide in average).
- 118,602 triplets were used for the training (approximately 90%) with a train/validation ratio of 90%–10%.
- 13,070 triplets were used for the test (approximately 10%).

Data augmentation was done during training to reduce overfitting and improve model generalization: random horizontal and vertical flips were applied to the triplet of images, and random adjustments to brightness, contrast, and saturation (±10%) were applied only to BF and IR images. All transformations were applied with a probability of 0.5.

**FIGURE 9:**
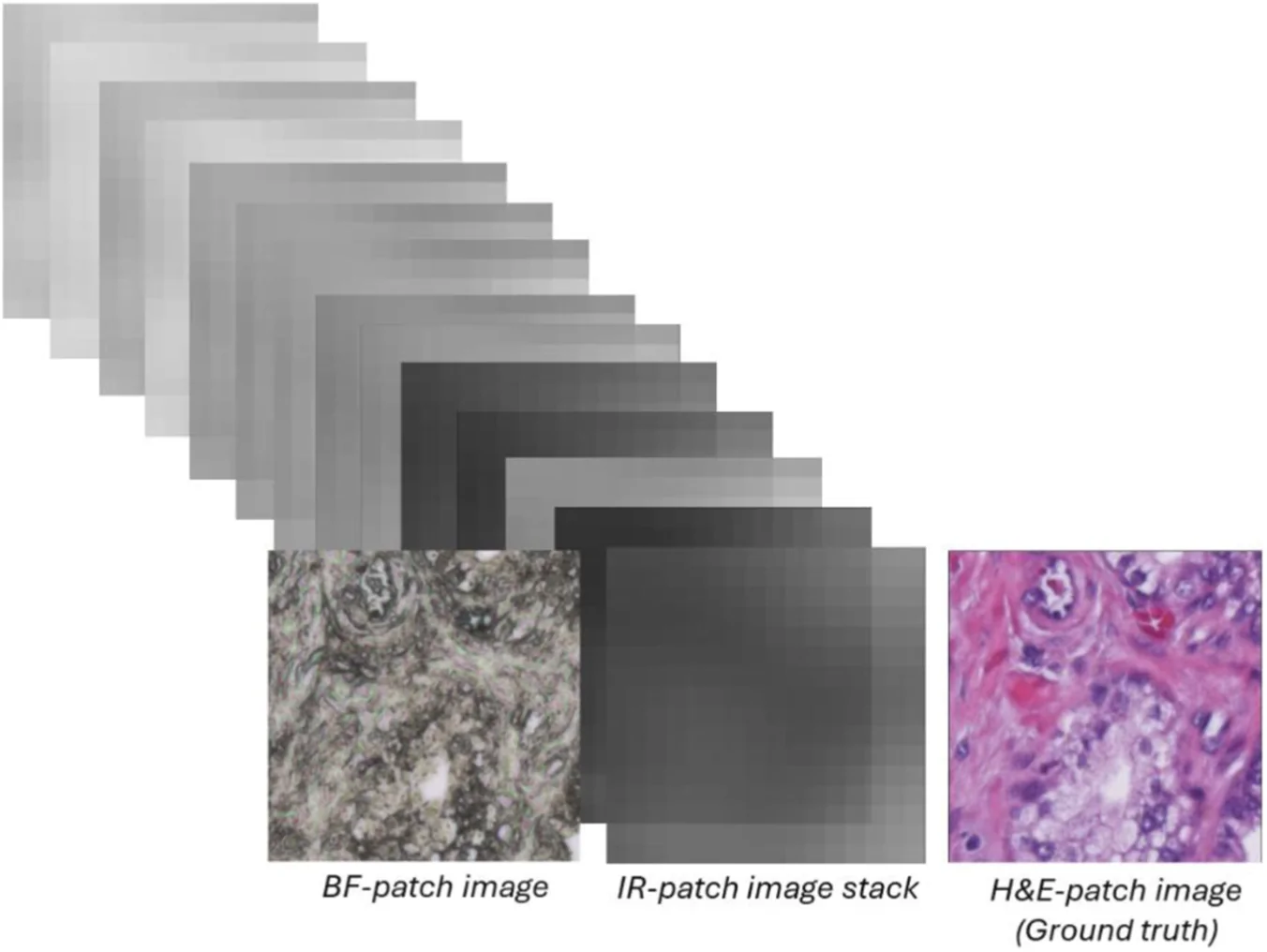
Typical triplet of patch images used for the training: from left to right: (256 × 256px) bright field image of unlabeled tissue, (16p×16px) IR-image stack of unlabeled tissue and (256p×256px) bright field image of H&E-stained tissue. The IR images are presented in grey scale: black areas indicating large absorption while the white areas indicate low absorption.

Representative image triplets are illustrated in Figure 9.

### 2.4 Preclinical evaluation–methodology

Beyond conventional mathematical metrics for image quality assessment, we conducted statistical evaluations of both perceived image quality and diagnostic performance using pathologist-derived ratings.

We selected 109 *ROIs* from prostate tissue sections that were unknown to our model. We applied our model to get 109 H&E inferred *ROI* images that were compared with their H&E ground truth counterparts. We finally got two sets of 109 images GT and IN (ground truth set and inferences). GT and IN in Equation 8 were randomly mixed up to have two complementary lists L and $\bar{L}$ composed of both inferred and ground true images, Equation 9.

$$\begin{array}{c} GT=\left\{GT_{1},\ldots ,GT_{109}\right\}\\ IN=\left\{{IN}_{1},\ldots ,{IN}_{109}\right\} \end{array}$$ (8)

$$\left(GT,IN\right)\overset{shuffle}{\to }\left(L,\bar{L}\right):L=\left\{GT_{1},{IN}_{2},GT_{3},\ldots ,{IN}_{109}\right\}\bar{L}=\left\{{IN}_{1},GT_{2},{IN}_{3},\ldots ,GT_{109}\right\}$$ (9)

**FIGURE 10:**
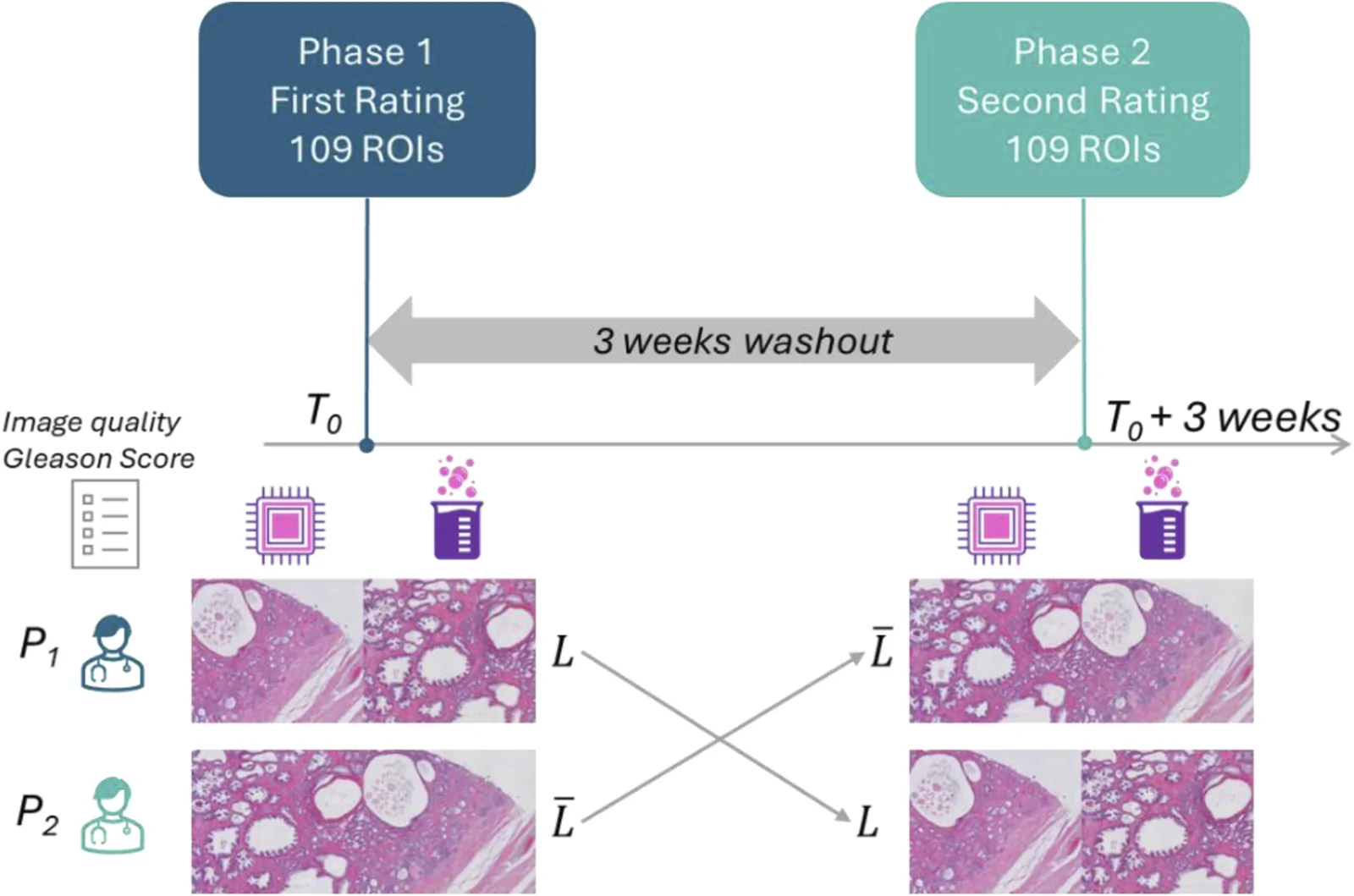
Preclinical assays method: We selected 109 ROIs from prostate tissue sections under test. To each ROI, it corresponds a pair of images (inferred image, ground truth) and we got two sets of 109 images (ground truth set and inferences). These two sets were randomly mixed up to have two complementary lists L and $\bar{L}$ composed of both inferred and ground true images. The two sets of images were sent to two pathologists (P1 and P2) for rating (image quality rating and Gleason scores). After 3 weeks of washout, the two lists were swapped between P1 and P2 for a second rating.

The evaluation was then performed in two phases: first L is sent to the pathologist $P_{1}$ while $\bar{L}$ was sent to $P_{2}$ for evaluation. After 3 weeks (the washout duration), $P_{1}$ evaluated $\bar{L}$ and $P_{2}$ evaluated L , see Figure 10. Pathologists rated the image quality based on criteria presented in Tables 1, 2, as defined by the French Association for Quality Assurance in Pathology – AfAQap (Egele, 2024). The Table 2 describes the notation (rating from 1 to 4) used in the rest of our study.

**TABLE 1:** Image quality criteria adopted for preclinical evaluation done on prostate tissue sections.

Image quality criteria	Comment
Nuclear components	The chromatin is distinct, with blue/purple staining
	The nucleolus is well-contrasted, dark blue-purple
	The nuclear membrane is clearly defined
Morphological characteristics of the cytoplasm	Good contrast compared to the extracellular matrix
	Eosinophilic granules are well-defined, with red-orange staining
Extracellular matrix components	Erythrocytes are bright red
	Collagen is yellow-orange in staining (with H&E); the fibrillar structure is apparent

**TABLE 2:** Meaning of the selected rating.

Rating	Comment
4/4 (Optimal)	Excellent, near perfect
3/4 (Good)	Good, with a few areas that could be improved
2/4 (Bad)	Average quality, but sufficient to establish a diagnosis or prognosis
1/4 (Unsuitable)	Insufficient, which may result in diagnostic or prognostic errors

The evaluation was then completed by statistics applied on the Gleason grade set on the ground truth and inferred ROIs.

This score is used by all pathologists to assess the aggressiveness of prostatic adenocarcinoma. It is calculated by adding the grade of the most common cancer pattern to the grade of the second most common pattern. Each grade ranges between 3 and 5 and the Gleason score ranges from 6 (3 + 3) to 10 (5 + 5) (Epstein et al., 2016; JLH van Leenders et al., 2020). The higher the score is, the worse the prognosis becomes. The rating used within this study is summarized in the Table 3.

**TABLE 3:** Rating used to perform the biostatistics.

Rating	Gleason Scores	ISUP	Meaning
0	Healthy	0	No cancer
1	IHC	NA	Doubt on the tissue integraty - IHC required to rise the doubt
6	Grade 6 (3 + 3)	I	Well-differentiated, uniform glands, minimal risk of aggressive behavior
7	Grade 7 (3 + 4)	II	Predominantly well-differentiated or moderately differentiated with focal moderate differentiation
	Grade 7 (4 + 3)	III	Predominantly moderately or poorly differentiated with minor component of well differentiated glands
8	Grade 8 (4 + 4)	IV	Poorly differentiated, cribriform or fused glands, significant risk of aggressive behavior
9	Grade (4 + 5 or 5 + 4)	V	Very high risk: Poorly differentiated or undifferentiated, high likelihood of metastasis
10	Grade 10 (5 + 5)		

## 3 Results and discussion

### 3.1 Qualitative results

**FIGURE 11:**
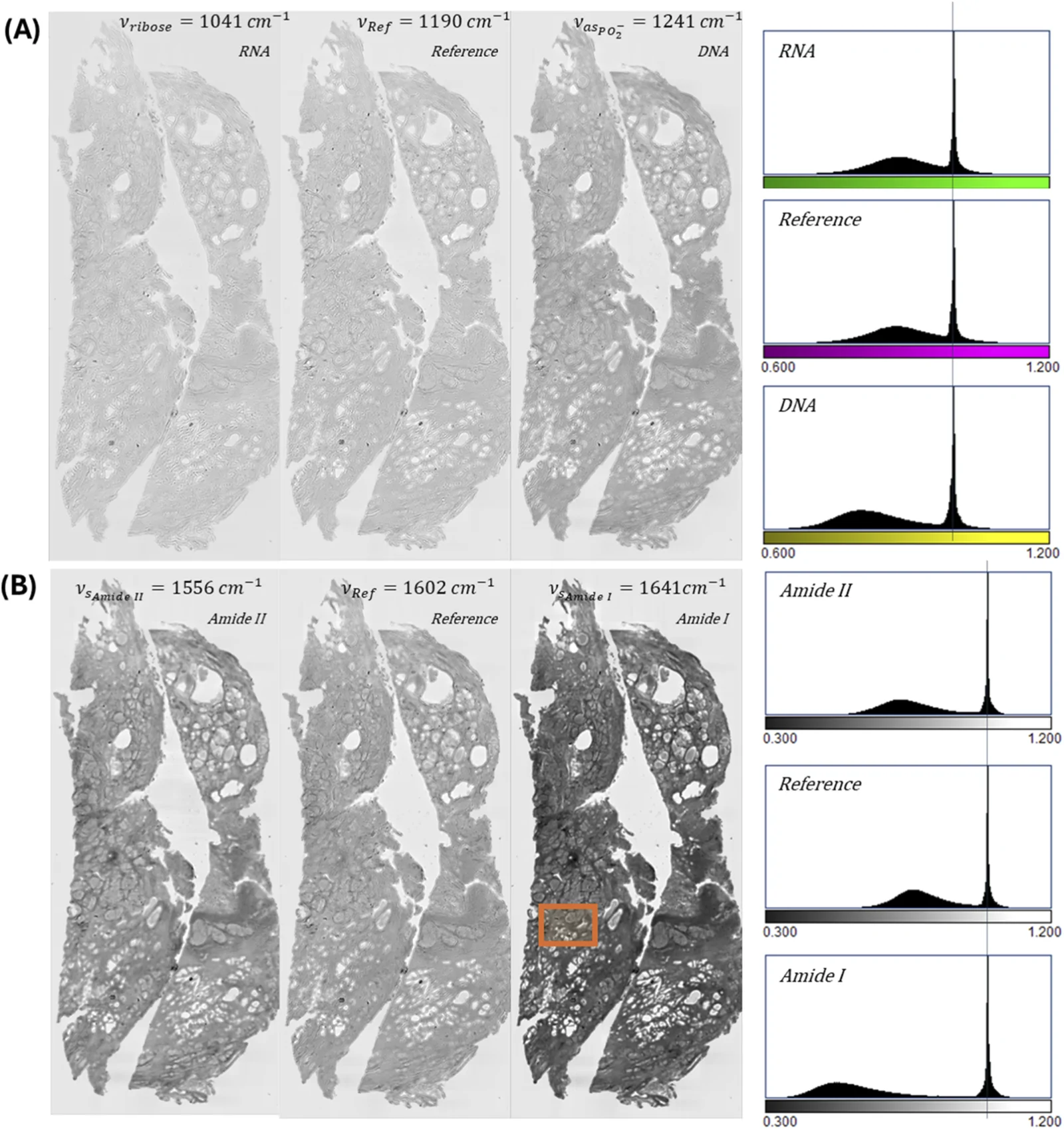
Whole slide images of a prostate tissue (2.74 × 2 cm^2^) (#B00980 – see SI) – **(A)** Infrared transmission of vibrational modes in nucleic acid, **(B)** Infrared image of amide vibrational modes in proteins: amide I and amid II – The corresponding histograms for each image are displayed on the right. – The orange box area is the ROI shown in the next figure.

Figures 11A,B present infrared images (2.74 cm × 2 cm) of the same unstained prostate tissue slide (#B00980; see SI), acquired at wavelengths targeting nucleic acids and proteins. These images reveal absorption contrasts corresponding to ribose (1,041 cm^-1^)-phosphate (1241 cm^-1^) vibrational modes and amide I-II (1,641 cm^-1^/1,556 cm^-1^) vibrational modes, respectively. The absorption band around 1,040 cm^-1^ reflects overlapping contributions from ribose-related RNA vibrations and carbohydrate C–O stretching. In prostate tissue, which is characterized by high epithelial cellularity and limited glycogen content, this band is expected to be predominantly influenced by RNA-associated vibrations. Distinct absorption contrasts are evident between the target and closest reference images at protein-specific wavelengths, while nucleic acid-associated contrasts, although less pronounced, remain sufficiently above noise and background levels—as demonstrated by the accompanying histograms—to be effectively used by the model. The histograms exhibit two distinct peaks: a sharp peak corresponding to the background (no tissue) and a broader peak associated with the tissue, and more specifically, with the probed biochemical bound.

**FIGURE 12:**
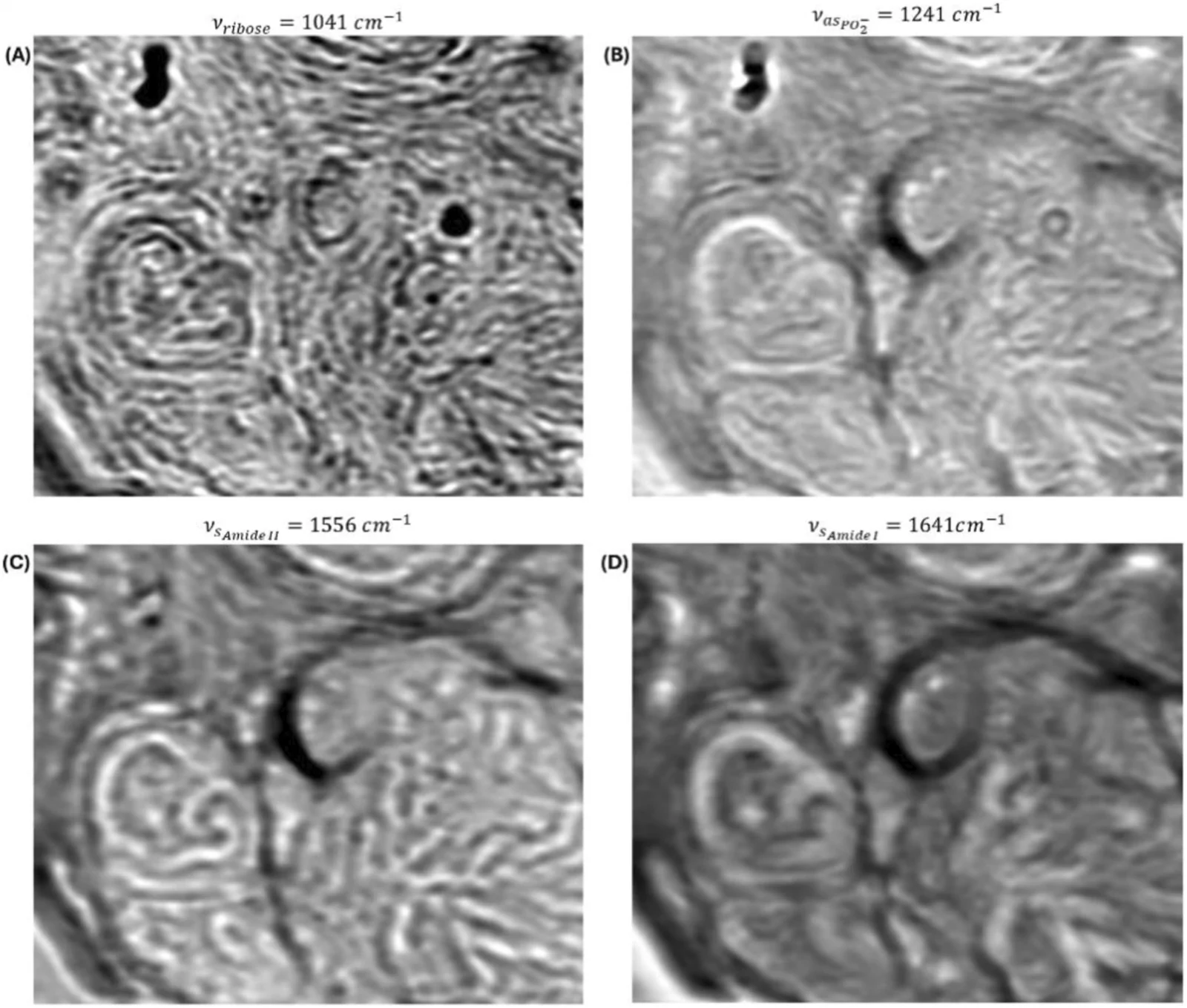
Images of the ROI delineated by the orange box in the previous figure (#B00980) – **(A–D)** From left to right: IR images of ribose in RNA, diphosphate in DNA, Amide II in proteins and Amide I (Helicoidal) in proteins.

Figure 12 depicts the ROI highlighted by the orange box in Figure 11. It displays IR images acquired at four key wavelengths: two targeting nucleic acids (A–B) and two targeting proteins (C–D). Diffraction artifacts, particularly at histological structure boundaries, result in blurred contours of larger features. Smaller structures, especially those below the 5 µm threshold, may exhibit reduced contrast. Additionally, diffraction-induced constructive interference can produce localized hot spots, requiring careful management of the bolometer’s dynamic range.

**FIGURE 13:**
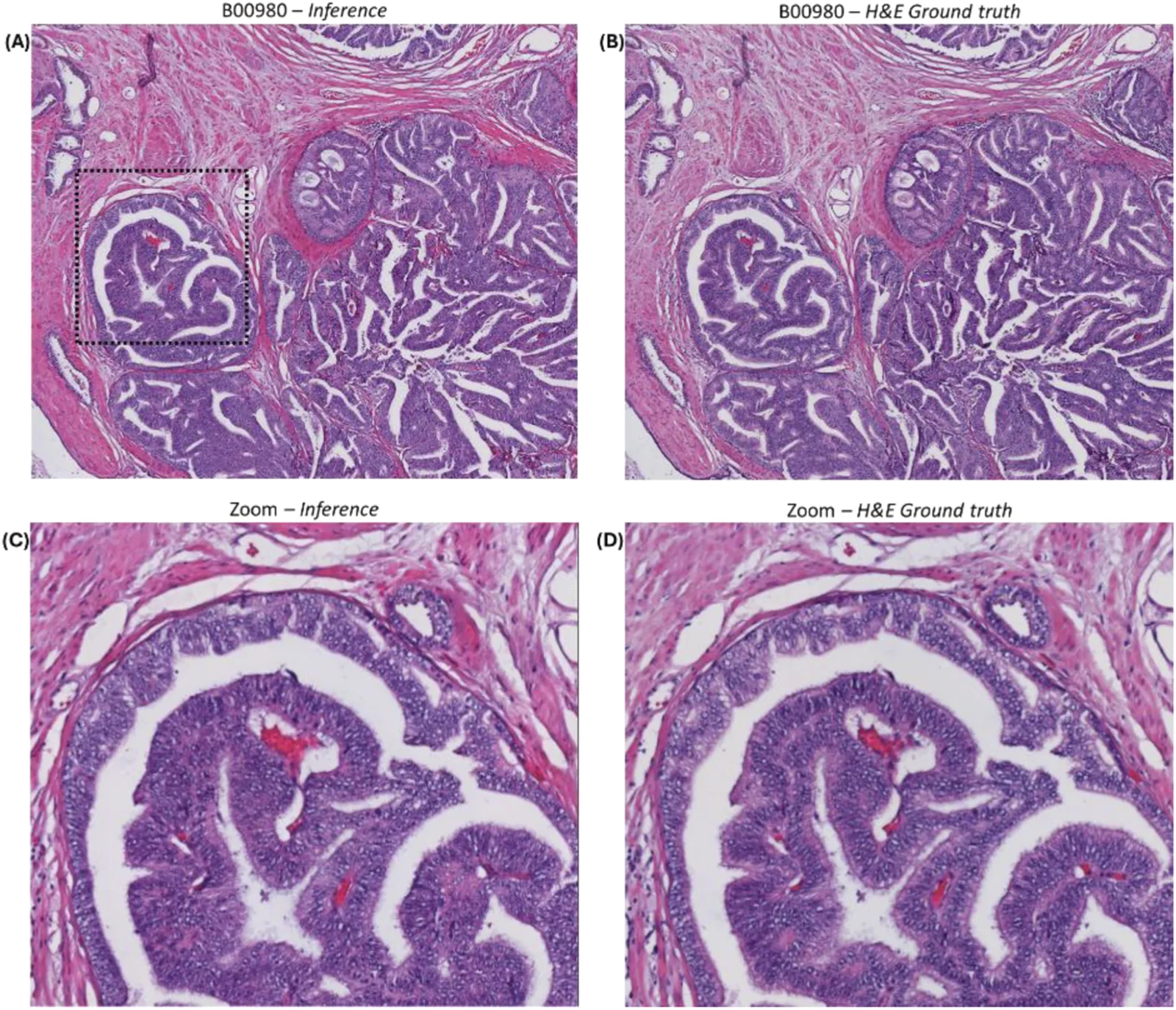
**(A)** Digital H&E inferred with our deep learning model of a ROI (#B00980) – **(B)** Current H&E staining of the same ROI – Ground truth – **(C)** Zoom on a gland area of the inferred ROI (dotted box) – **(D)** Zoom of the same area of the H&E stained tissue.

**FIGURE 14:**
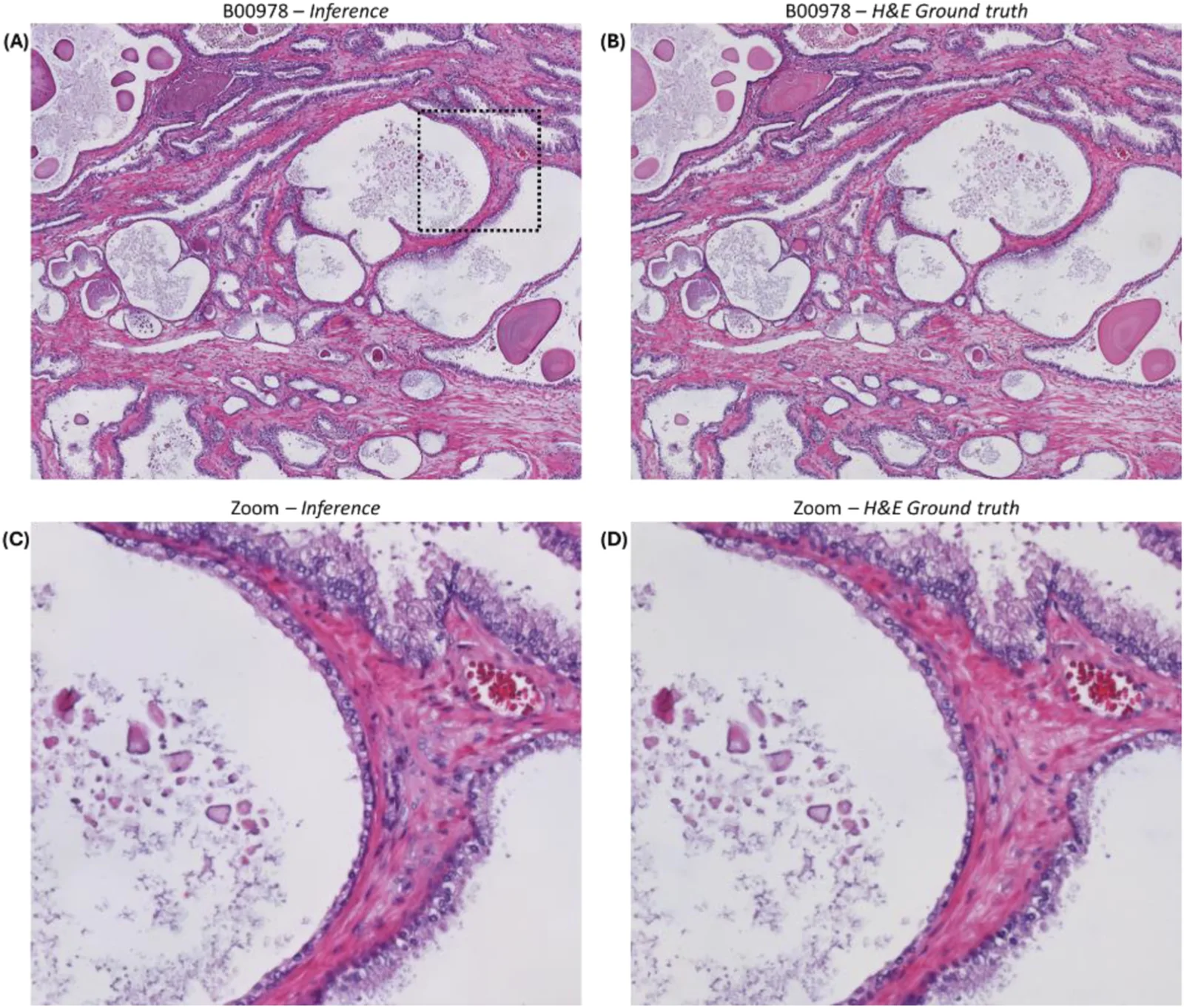
**(A)** Digital H&E inferred with our deep learning model of a ROI (#B00978) – **(B)** Current H&E staining of the same ROI – Ground truth – **(C)** Zoom on a gland area of the inferred ROI (dotted box) – **(D)** Zoom of the same area of the H&E stained tissue.

Figures 13, 14 present comparisons between the true and digital H&E staining for two ROIs from two tissue sections (#B0098 and #B00978). It is important to note that the ROIs were realized from the same tissue, not adjacent tissues, ensuring full comparability. From left to right, the digital H&E generated by our model and the ground truth H&E are shown. Notably, cytological structures as well as the color contrast in the digital H&E image, are faithfully reproduced and resemble those observed in the true H&E staining. In particular, the shape of cells, nuclei, glands, vascular structures, and their contents, as well as connective tissue, are quite well reproduced. The colorimetry is also comparable to chemical H&E staining.

The single high-resolution bright-field (BF) image enables the extraction of multi-scale morphological features, ranging from single-pixel and local pixel clusters to macro-textural patterns. Complementing this, the low-resolution 16-image infrared (IR) stack provides localized spectral fingerprints at the IR pixel resolution of 8 µm (The 256x256 pixel patch in the BF image maps directly to a 16x16 pixel region in the IR domain). Because each IR pixel represents a spatial and spectral average across approximately one to two cells, the model is trained to leverage these IR signatures alongside key biochemical ratios (e.g., PO_2_-DNA to PO_2_-RNA, PO_2_-DNA to Amide I…) These distinct spectral bands and intensity ratios provide critical, complementary information that drives model performance.

### 3.2 Quantitative results–metrics

Beyond the qualitative comparisons, we have considered four quite common metrics computed in image processing to measure image similarity including, from the simplest to the most complex in terms of computation complexity: Peak Signal-to-Noise Ratio ( PSNR ) (Oktay et al., 2018), Structural Similarity Index Measure ( SSIM ) (Horé and Ziou, 2010), Multi-Scale Similarity Index Measure ( MS−SSIM ) (Wang et al., 2003; LPIPS Zhang et al., 2018).

SSIM and PSNR are widely used in the digital pathology community. Here, we also added the MS – SSIM metric that is less sensitive than SSIM to pixel shifts that can occur during the acquisition and that have been not fully corrected in the registration phase—for more information, the pixel shift impact on the SSIM and MS−SSIM is presented in the SI. LPIPS is also well suitable for a human perception based comparison. Ideally, the SSIM and MS−SSIM should reach a value of 1 (arrow up), *PSNR* should tend to +∞ and LPIPS should tend to 0 since it corresponds to a distance between the ground truth features and the inferred counterparts (0 means the two images are considered as identical by the neuron network). ROI backgrounds were excluded from metrics computations. This avoids any bias that would lead to an artificial overestimation of the performance (in particular of the SSIM and PSNR metrics). For more information on the overall data treatment process, please refer to the SI.

Metrics computed on the test dataset lead to quantitative results: $SSIM=0.78\;\left(\pm 0.057\right)$ , $MS-SSIM=0.82\;\left(\pm 0.072\right)$ , $PSNR=23.71\;\left(\pm 2.454\right)$ and $LPIPS=0.039\;\left(\pm 0.039\right)$ . PSNR and SSIM are reported in Table 4, which also compiles the results of the most representative studies from the literature applied to virtual H&E staining. Although experimental context and conditions (organ, resolution, acquisition type, test database size and test set preparation) may differ from our work, it still offers a literature baseline to compare our work. We excluded from this table any paper reporting a small number of test ROIs, applying pretreatment such as blurring or presenting results from a validation dataset, as these strongly overestimate performances. We intentionally did not include results obtained using complex spectroscopic approaches, such as photoacoustic or stimulated Raman imaging (Sarri et al., 2019; Boktor et al., 2024; Liu et al., 2024; He et al., 2026). Although these methods are scientifically compelling, in our view, their technical complexity currently limits their adoption in routine laboratory settings.

**TABLE 4:** Comparison of the metric values obtained with our model with values reported for some works from the literature.

Inputs	M	Model	Tissue	SOST​	# test patches	SSIM (↑)​	PSNR (↑)​	LPIPS (↓)​	BSA	Reference
IR + Brightfield	x20​	MUGAN	Prostate	yes	13,073	0.78	23.71	0.236	yes	This paper
Autofluorescence	x40​	RegiStain	Autopsy lung	yes	∼96,100	0.82	20.32	-	-	Li et al. (2024)
Autofluorescence	x40	Brownian bridge diffusion	Lung	yes	∼2,530	0.69	18.52	0.27	-	Zhang et al. (2025)
Brightfield	x40​	pix2pix	Murine Prostate	yes	∼90,000	0.746	22.865	-	yes	Khan et al. (2023)
Brightfield	x20​	pix2pix	Prostate	no	∼214,960	0.902	22.82	-	-	(Rana et al. (2020))​

M, magnification; SOST, Strict out-of-sample testing; BSA, Background subtraction applied.

We emphasize that this comparison provides a useful overview of current performance levels but should be interpreted with caution, as the results were obtained at different magnifications (×20 and ×40), and the reported number of patches is approximate, since other studies do not necessarily compute metrics on 256 × 256-pixel patches. Notably, image registration, which strongly affects SSIM and PSNR , is even more critical at ×40 magnification.

In our case, PSNR and SSIM exhibit pretty high values. The PSNR values (typically ∼ 23), are above values reported in the Table 4. The SSIM values of approximately 0.78 are already satisfactory since the MS−SSIM values, which is less pixel-shift sensitive, reach up to 0.84 (note reported in the Table 4) that is indicative of strong inference performance. These SSIM and MS−SSIM performances suggest that our network maintains cell/tissue architecture and structure fairly well. Our model’s performance underscores the broader efficacy of cross-scale, bi-modal data fusion. Integrating the IR spectral stack with the BF data yielded a measurable performance increase, raising the SSIM from 0.746 to 0.780 and the PSNR from 22.87 dB to 23.71 dB compared to a BF-only baseline. Notably, the metrics obtained using the BF image alone are in close agreement with values reported in existing literature (Khan et al., 2023). Furthermore, these IR + BF metrics are expected to be enhanced in future iterations by optimizing the physical optical setup, in particular removing spurious interference patterns. LPIPS values obtained by our staining model are within a good range and suggest that morphological structures are likely preserved and there are only moderate perceptual differences between our inferred H&E and the ground truth. Our results are very encouraging in the context of the literature, they are better than other conditional GAN-based work, and competitive to more recent diffusion-based methods.

The autofluorescence-based approach is close to our methodology in terms of tissue handling and image preparation. The registration component in (Li et al., 2024), which is similar to the VoxelMorph framework (Balakrishnan et al., 2019), appears, in our view, to be slightly more efficient than our approach and results in a marginally higher SSIM . As a minor additional point (Ma et al., 2026), benchmarked their registration strategy across several models (Pix2pix, GAN-based methods, BCI, etc.) on the AF2HE dataset (15 breast/lung WSIs, 10× autofluorescence), reporting PSNR and SSIM gains of 5.3% and 4.8% (27.8 dB and 0.766, respectively) attributable to registration alone. This offers a useful, if tangential, illustration of how registration affects image-quality metrics. In the brightfield-based approach described by (Rana et al., 2020), samples used for training are reused for testing, which, in our view, leads to a clear overestimation of performance. Moreover, we assume that the image background is retained, which would further inflate the reported metrics. Finally, the registration performed using Photoshop results in the SSIM value that appears unexpectedly high–especially considering such a large number of images.

### 3.3 Statistical analyses–preclinical validation

The reported MS – SSIM and SSIM values should only be interpreted as quantitative indicators of morphological fidelity. Beyond these indicators, we went a step further and conducted a preclinical evaluation led by two pathologists regarding the image quality rating and diagnosis/prognosis scores. This pathologist-based assessment provides complementary evidence of diagnostic reliability.

#### 3.3.1 Statistical analyses on image quality scores

The objective of the first analysis is to determine whether our multimodal infrared digital staining can achieve the same image quality as H&E chemical staining.

**FIGURE 15:**
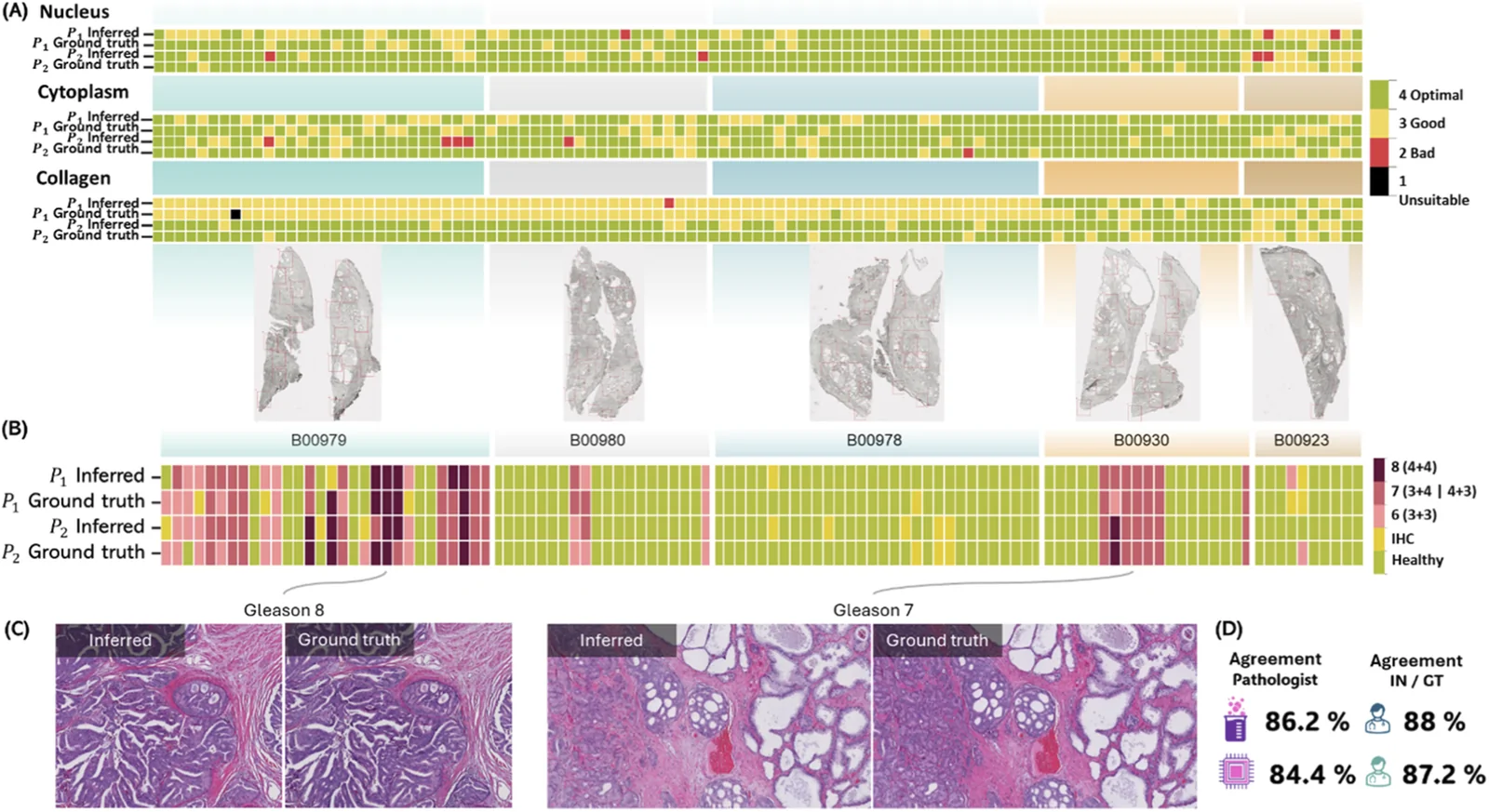
**(A)** Rating of the image quality done by the two pathologists including components: nucleus, cytoplasm and extracellular matrix (i.e. collagen), see Table 1 – Each of square column corresponds to a ROI – **(B)** Grading set by pathologists for the 109 ROIs (in their inferred and Ground truth versions): Comparison according to ROI – each column of square corresponds to a ROI – **(C)** Typical ROIs at different Gleason score s– **(D)** Comparison by pathologist and agreement percentages (intra-pathologist and Inferences versus Ground Truth).

The results of quality ratings carried out by the two pathologists are summarized in Figure 15A. At first sight, there is a good agreement between inferred image quality and image quality of ground truths. The ratings are mainly between 3 and 4 demonstrating the good overall quality of colors and morphologies of tissues reproduced by our approach. The majority of low scores (<3) were attributed to inferred ROIs (10/12 cases), indicating some room for improvement, particularly in the rendering of nuclear components. In most ROIs, $P_{1}$ considered that the collagen appeared a bit too red and set a score of 3. The Table 5 gives the percentage of ROIs considered optimal (rating = 4) and good (rating ≥ 3 in brackets) for diagnosis or prognosis for the three main cellular elements in the ground truth and inferred ROIs—see Table 1 for the selected criteria. These results show that the digital rendering of nuclear pixels can be further optimized, even when pathologists deem the current image quality sufficient for diagnostic purposes. Notice that near 100% of digitally stained ROIs received a score of 3 (good) for the three categories.

**TABLE 5:** Percentages of ROI considered as optimal (score = 4) by the two pathologists for the three cellular components: comparison between ground truth and digital staining – In parentheses, the percentage of ROIs with a score larger or equal to 3 (good).

Optimal image Rating=4 ( ≥3 ) tissue elements	$P_{1}$		$P_{2}$	
	Ground truth	Digital staining	Ground truth	Digital staining
Nucleus	87% (100%)	67% (97%)	90% (100%)	81% (96%)
Cytoplasm	81% (100%)	72% (100%)	90% (99%)	72% (95%)
Collagen	15% (99%)	18% (99%)	87% (100%)	86% (100%)

As shown in Table 6, we applied the χ ^2^ two-sided test of homogeneity (Ground truth versus inferred) (Faizi and Alvi, 2023) on the image quality scoring with null hypothesis $H_{0}:p_{GT}=p_{IN}$ for each cellular components (nucleus, cytoplasm and collagen) to objectively quantify the difference between inferred and ground truth ROIs. It turns out that there is rejection of homogeneity between ground truth and digital scores for nucleus for $P_{1}$ and for cytoplasm for $P_{2}$ (5%- significance level). For other elements, ground truth ROI quality and inferred ROI quality are not statistically differentiated, with a quality of collagen equivalent for both pathologists. While these preliminary results suggest that our digital staining could perform comparably to the gold-standard chemical H&E stain, a larger sample size is required to definitively conclude its equivalence and fully establish the model’s robustness for clinical pathology.

**TABLE 6:** Stain quality scoring – Statistical analysis with the χ ^2^-test.

Two-sided test between ground truth and inferred	Nucleus		Cytoplasm		Collagen	
​	$P_{1}$	$P_{2}$	$P_{1}$	$P_{2}$	$P_{1}$	$P_{2}$
$\chi^{2}\;p-value$	0.0012*	0.0544	0.1121	0.0025*	0.4693	0.8420

* 5% significance level

#### 3.3.2 Statistical analyses on the gleason scores

The objective of this study is to answer the question: are digitally stained slides equivalent to H&E chemically stained images for prostate cancer diagnoses and prognoses?

The gradings of every ROIs are presented in the Figures 15B,C. To establish the statistic, we have also added the category “IHC” in the event that the pathologist cannot set a definitive diagnosis that would require a complementary immunohistochemistry analysis. The intra-pathologist agreement is 86.2% for ground truth ROIs and 84.4% for inferred ROIs. The agreement between chemical H&E staining and our digital staining is 88% and 87.2% for $P_{1}$ and $P_{2}$ respectively (Figure 15D). The model achieved an 88% average agreement with the ground truth, slightly surpassing the typical 85% intra-pathologist agreement rate (15% human discordance). Nevertheless, testing across a larger cohort is essential to definitively confirm these performance gains.

We applied the χ ^2^ two-sided test of homogeneity (Ground truth versus inferred) on the Gleason scoring with null hypothesis $H_{0}:p_{GT}=p_{IN}$ (5%-percent confidence level). The statistics are presented in Table 7. In our assessment of Gleason scores, the hypothesis test is not significant, demonstrating that pathologists were unable to distinguish between synthetic and chemical staining. With a 95% confidence interval, this result confirms that digital staining may be diagnostically equivalent to chemical staining for prostate grading. Diagnoses or prognoses based on observation of images digitally stained by our DL model seem to be as meaningful as those based on chemically stained tissue images. Complementary elements that confirm the results are shown in SI.

**TABLE 7:** Gleason grading – Statistical analysis with the χ ^2^-test.

Two-sided test between ground truth and inferred	Gleason	
	$P_{1}$	$P_{2}$
$\chi^{2}\;p-value$	0.890	0.515

## 4 Conclusion and perspectives

We have introduced a fast bimodal microscopy platform combining brightfield and infrared imaging, along with a deep learning-based optical staining model capable of processing both modalities. Conceptually, this bi-modal approach mirrors a well-established paradigm in astrophysics, most notably exemplified by the James Webb Space Telescope, which routinely synthesizes high-spatial-resolution near-infrared structural data with lower-resolution mid-infrared spectral bands to maximize global scene information. In a similar vein, our architecture successfully leverages the crisp, sub-micron morphological boundaries of the BF image to structurally contextualize and resolve the coarse-grained biochemical pixels (8 µm) of the IR stack.

Our results demonstrate strong agreement between our digital staining method and the chemical H&E staining reference. Notably, our approach excels in accurately rendering nuclear and nucleolar structures. We have demonstrated our capability to get IR-WSI over 5 cm^2^ tissue surface in few minutes making this approach fully compatible with pathology workflow constrains.

The automatic registration and the deep learning staining model have led to pretty good metrics on test WSI samples (over 23 for PSNR , and close to 0.8 for SSIM and MS−SSIM ). The performance metrics obtained with our IR-based digital staining even surpass those reported in the literature. To further benchmark our digital imaging approach against the histopathological gold standard, a preclinical validation study, conducted in collaboration with expert pathologists, has then been presented. Within this preclinical validation framework, preliminary statistical analyses based on morphological criteria, such as nuclear, cytoplasmic, and extracellular features, and Gleason grading have been provided. While these metrics offer an initial, clinically meaningful assessment of our model’s performance, evaluation on a larger cohort remains necessary to definitively confirm these findings.

Our IR-based digital staining achieves image quality sufficient to support equivalent diagnostic and prognostic assessments as those performed on conventionally stained slides. The image quality scoring of digitally stained ROIs has been considered good enough by pathologists, sometimes even optimal. Most of the time, the statistic has demonstrated that the scores obtained from the digital images cannot be distinguished from the images obtained from current H&E staining (the agreement between inferred and ground truth ROIs reached 88%). However, detailed analysis of ROI images–systematic observations and comparisons of images—and statistics show that nucleus rendering, in terms of number and details, can still be improved. We are currently working on enhancing IR-spatial resolution to enhance nuclei detection in particular. In addition, we are improving the architecture of the DL model by replacing the CNN by a visual transformer architecture for the Generator.

The DL model developed for prostate tissues serves as a basic model that we are generalizing to colon, breast and skin tissues. H&E staining relies on universal acid-base chemical reactions, enabling consistent staining across all organs using the same underlying chemistry. Similarly, IR imaging reveals universal biochemical information that is intrinsic to the tissue. We therefore believe this approach will yield a digital staining model generalizable to any organ and cancer type. The infrared-derived features are expected to enhance this task by introducing novel metabolomic insights not captured in prior approaches.

The IR-setup presented in this paper is also being embedded into a bimodal IR-VIS-scanner with some modification on the IR-optical path to gain in compactness. A first prototype is shown in the Figure 1.

Looking forward, this infrared-driven optical staining approach will be further advanced through the integration of metabolomic maps – such as collagens, lipids, carbohydrates, and selected proliferation markers – overlaid onto H&E-like images (Kallenbach-Thieltges et al., 2020; Gerwert et al., 2023). Ultimately, this work lays the foundation for broader adoption of infrared spectroscopy in pathology laboratories, facilitating the identification of advanced diagnostic and prognostic markers based on complex IR molecular signatures that will be superimposed to our digital H&E images.

## Data Availability

The raw data supporting the conclusions of this article will be made available by the authors, without undue reservation.

## Footnotes

1 In QPI techniques we include Phase contrast or differential interference contrast (DIC).

2 Broadband means that the camera maintains consistent sensitivity across the entire useful infrared wavelength range.
