# Supplementary material for: Mid-infrared microscopy for label-free digital staining: initial clinical assessment
Source: Front Bioeng Biotechnol. 2026 Aug 31;14:1774279. doi: 10.3389/fbioe.2026.1774279 (PMC13572712; doi:10.3389/fbioe.2026.1774279)
Supplement: Supplementary file 1 [file Supplementaryfile1.pdf]

Supplementary Information

Mid-Infrared Microscopy for Label-Free Digital Staining: Initial Clinical Assessment

L. Duraffourg[1*], H. Borges[1], M. Fernandes[1], M. Beurrier-Bousquet[1], J. Baraillon[1], B. Taurel[1], J. Le Galudec[1], K. Vianey[2], C. Maisin[2], L. Samaison[1], F. Staroz[1], M. Dupoy[1]

[1]ADMIR, 137 rue Mayoussard, Bat C, 38430 Moirans, France

[2] Cypath-RB, 201 Route de Genas, 69100 Villeurbanne, France

*Corresponding Author: laurent.duraffourg@admir-analysis.com

1 Tissue sections

The study has been conducted on 15 tissue sections of prostate, see Table 1 and Table 2. Ten sections have been used for training. Five other sections have been used for the test of the AI model and the clinical validation. The sections are presented in the Figure 1 and Figure 2.

# Reference	Thickness (µm)	ORGAN	Pathology	Training / test	Number of ROIs	Number of patches
B00923	3	PRO	Adenocarcinoma	TEST	10	846
B00924	3	PRO	Adenocarcinoma	TRAINING	20	13441
B00925	3	PRO	Adenocarcinoma	TRAINING	20	16880
B00926	3	PRO	Adenocarcinoma	TRAINING	20	15728
B00927	3	PRO	Adenocarcinoma	TRAINING	15	13045
B00928	3	PRO	Adenocarcinoma	TRAINING	15	10383
B00929	3	PRO	Healthy tissue	TRAINING	20	15117
B00930	3	PRO	Adenocarcinoma	TEST	19	2845
B00931	3	PRO	Healthy tissue	TRAINING	15	7017
B00932	3	PRO	Healthy tissue	TRAINING	15	7583
B00933	3	PRO	Healthy tissue	TRAINING	20	12950
B00934	3	PRO	Adenocarcinoma	TRAINING	15	6458
B00978	3	PRO	Adenocarcinoma	TEST	30	3498
B00979	3	PRO	Adenocarcinoma	TEST	20	2067
B00980	3	PRO	Adenocarcinoma	TEST	30	3814

Table 1. Section samples used for the training, test and preclinical validation

	ROI	*Patches*	*Ratio*
TOTAL TEST	109	13070	9,93%
TOTAL TRAINING	175	118602	90,07%
TOTAL	284	131672	100,00%
Average patch number per ROI			463

Table 2. Number of ROIs and patch images – Training, test and preclinical evaluation

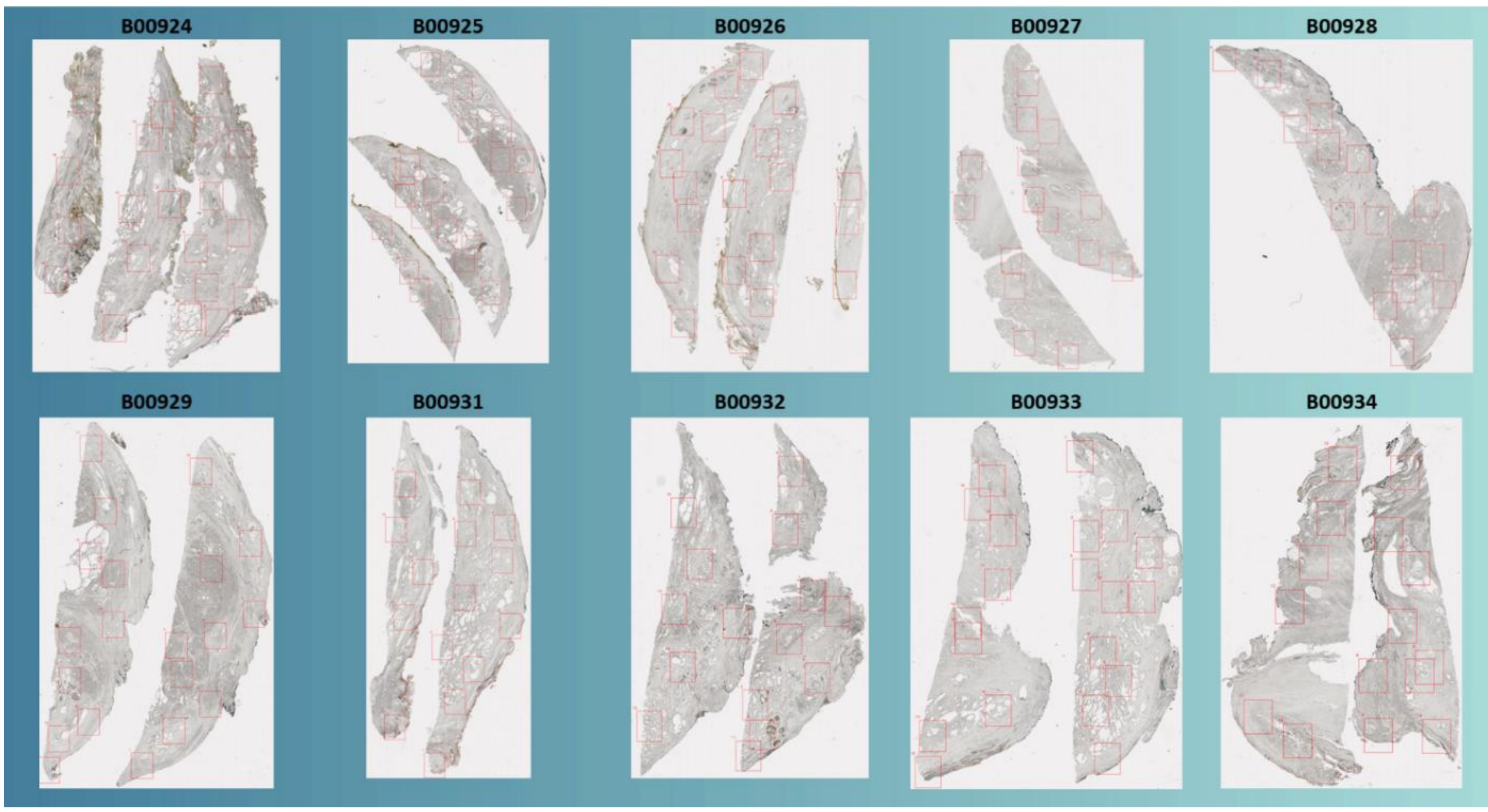

Figure 1. Prostate sections used for the training – Red squares are the ROI in each tissue section – ROIs are chosen to obtain an accurate representation of the tissue

This is a provisional file, not the final typeset article

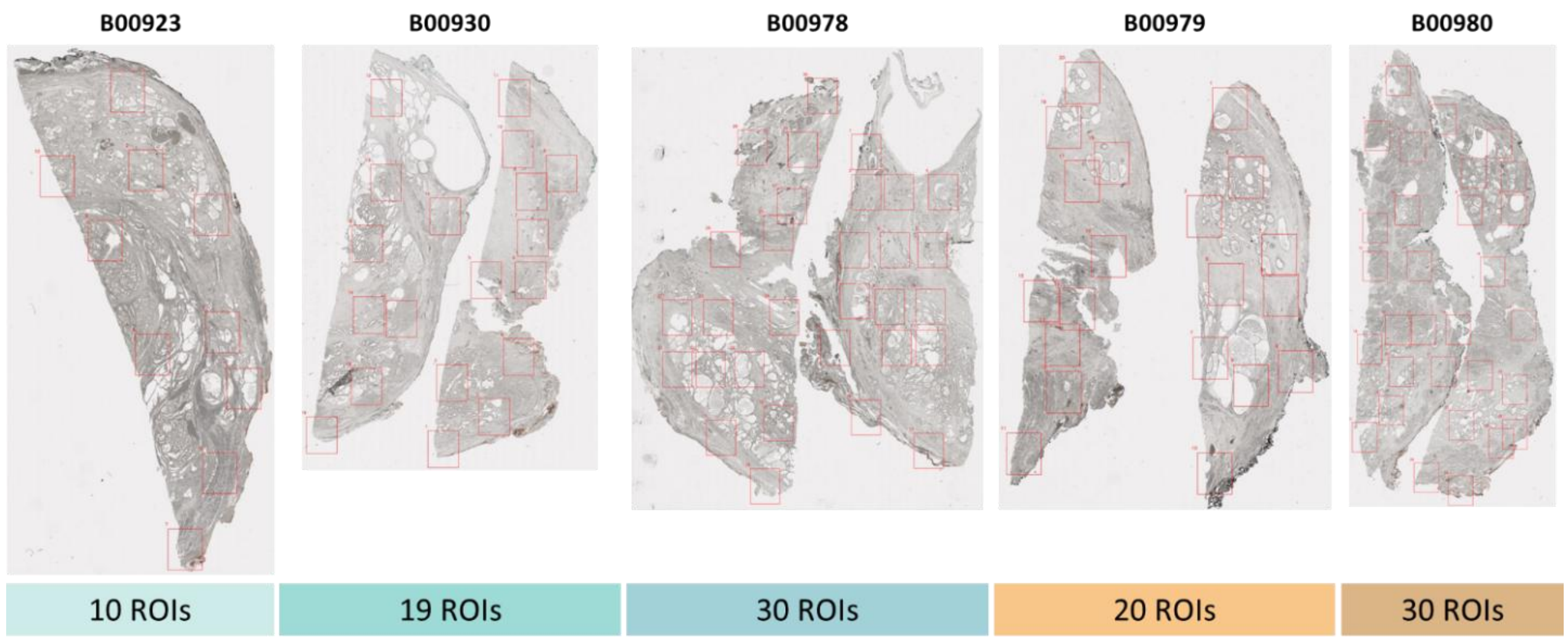

Figure 2. Prostate sections used for AI model testing and preclinical validation

2 Optical setup

The synopsis of the infrared scanner is shown in Figure 3.

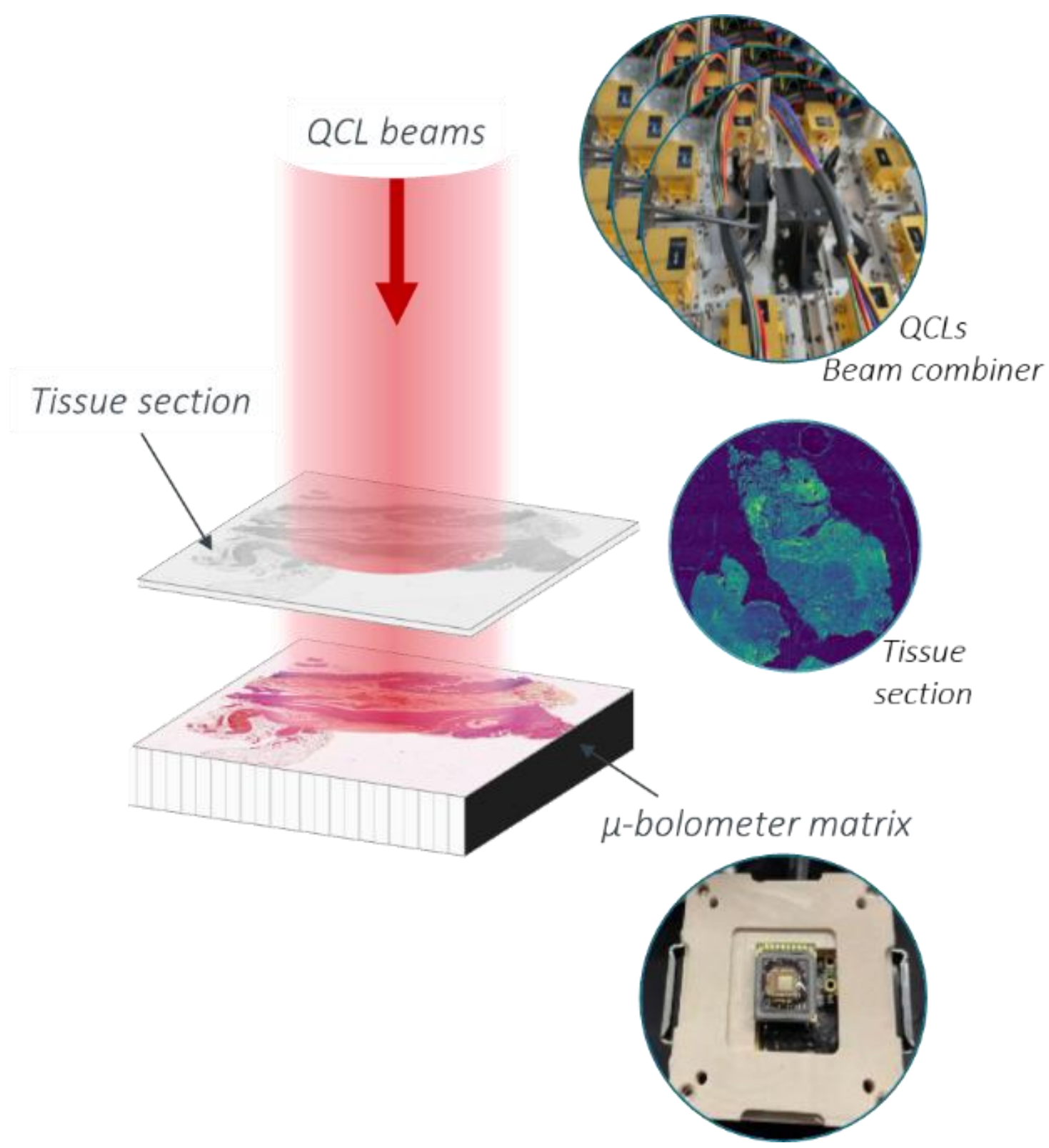

Figure 3. Synopsis of the Mid-IR microscope

Our IR source is composed of sixteen quantum cascade lasers (Coutard et al., 2020; Faist et al., 1994) whose wavelengths correspond to the rovibrational energies of specific chemical bonds in DNA, RNA, proteins, carbohydrates (Baker et al., 2014; Barth, 2007; Zhizhina & Oieinik, 1972). The wavelengths considered within this study are presented in the Table 3 and Table 4.

3 Main Infrared Absorption Bands Used in IR Whole-Slide Imaging

The QCL wavelengths were chosen from basic spectroscopic consideration for biological tissues, please refer to the Table 3 and Table 4 below.

Wavenumber (cm^{-1})	Dominant bond / group	Vibration type	Biomolecule	Relevance in IR-WSI
**3500–3200**	O–H / N–H	δ	Water, proteins	Usually excluded (water-dominated)
**3010**	=C–H	δ	Unsaturated lipids	Lipid unsaturation
**2960**	CH_3	δ_{as}	Lipids, proteins	Membrane composition
**2920**	CH_2	δ_{as}	Lipids	Lipid chain order
**2850**	CH_2	δ_s	Lipids	Lipid content
**1740**	C=O (ester)	δ	Lipids	Phospholipids, triglycerides
**1700–1600**	Amide I (C=O)	δ	Proteins	Secondary structure, cellularity
**1650**	Amide I maximum	δ	Proteins	Core protein marker
**1580–1510**	Amide II (N–H, C–N)	$\nu + \delta$	Proteins	Tissue architecture
**1450**	CH_2 / CH_3	ν (scissoring)	Lipids, proteins	Cell packing
**1400**	COO^-	δ_s	Proteins	Amino-acid side chains
**1245-1220**	PO_2^-	δ_{as}	Nucleic acids DNA>RNA	Nucleic-acid backbone
**1085-1070**	PO_2^-	δ_s	Nucleic acids DNA≈RNA	Nuclear density
1060–1040	C–O–P / ribose	δ (weak)	RNA (ribose) + carbohydrates	RNA-rich cytoplasm
1050–1020	C–O / C–O–C	δ	Carbohydrates (± RNA)	Glycogen, glycoproteins
970–960	C–O–P	δ	DNA + carbohydrates	Deoxyribose–phosphate

Table 3. Mid-infrared wavelengths of the infrared source – s : symmetrical, as : antisymmetric, δ: stretching mode, ν : bending mode

- The amide I and II are the dominant and most reliable protein markers
- We have intentionally excluded amide III, as it is weak/overlapping in IR and better accessed via Raman spectroscopy.
- The IR regions 3000-2800 cm^{-1} and 1800-900 cm^{-1} constitute the core spectral windows used in tissue.

This is a provisional file, not the final typeset article

- The fingerprint region (1200–900 cm^{-1}) is critical for machine-learning-based tissue classification and metabolic contrast.
- PO2- are the dominant and most reliable nucleic acid markers
- The region 1150-1000 cm^{-1} is globally dominated by carbohydrate absorptions

These bands reflect biochemical composition, enabling stain-free digital pathology. While absolute absorption intensities depend on section thickness and preprocessing, distinct tissue types exhibit reproducible relative absorption patterns in key IR bands, reflecting their underlying biochemical composition.

**Tissue**	**Lipid bands (3000–2800 cm^{-1})**	**Ester C=O (1740 cm^{-1})**	**Amide I–II (1700–1500 cm^{-1})**	**Phosphate (1240/1080 cm^{-1})**	**Carbohydrate (1150–1000 cm^{-1})**	**Notes**
**Breast**	+++	++	++	+	+	High adipose content
**Prostate**	**++**	**+**	**+++**	**++**	**+**	**Glandular, protein-rich**
**Skin**	+	–	+++	+	+	Keratin-dominated
**Colon**	+	–	++	++	+++	Mucins, glycans
**Lung**	+	–	++	++	+	Cellular heterogeneity
**Head & Neck**	+	–	+++	++	+	Squamous epithelium
**Brain**	+++	++	++	+	+	Myelin-rich (lipids)
**Liver**	++	+	++	++	+	Metabolic organ
**Muscle**	+	–	+++	+	–	Protein-dominated
**Stroma / ECM**	–	–	++	–	+++	Collagen, proteoglycans

Table 4. Relative IR Absorption Strengths by Tissue Type – Absorption strength : +++ strong, ++ moderate, + weak, – negligible (blue line for the prostate)

4 Registration method – Performance evaluation

To estimate the performance of the self-registration, we evaluated the target registration error by computing the distance between the self-registered landmarks (of the BF-H&E ROIs) and the original landmarks (of the unlabeled BF ROIs). The method is shown in Figure 4. The results obtained from three test WSI (corresponding to 7 ROI, 19 ROIs and 29 ROIs) are shown in Figure 5. Only 12 out of 204 landmarks (3 test WSI) exhibited errors exceeding 1 µm (greater than 2 pixels); these outliers occurred primarily because the landmarks were in tissue-free background regions.

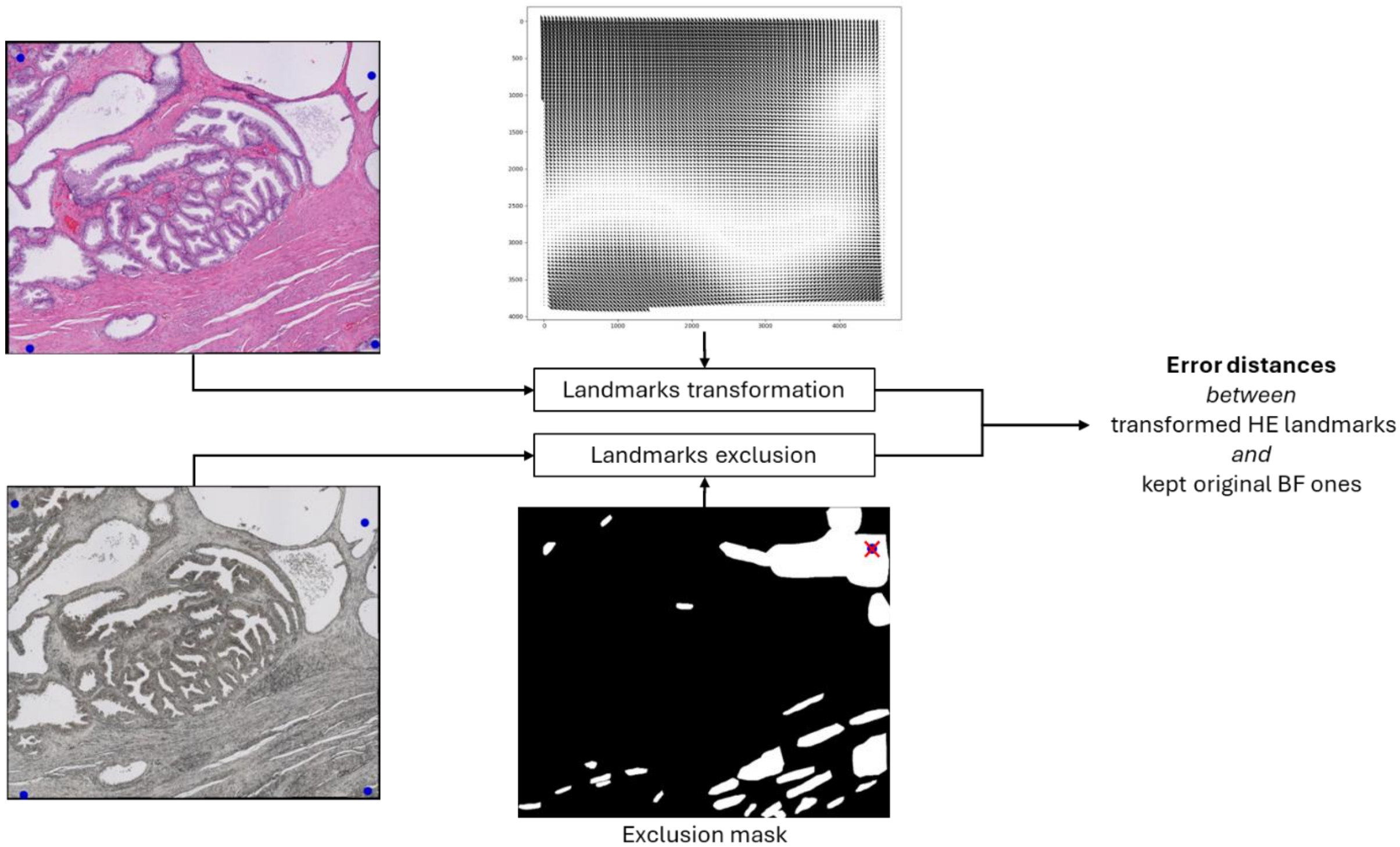

Figure 4. Method to estimate the registration error – We considered 4 landmarks (preferably) in the four corners of the BF-HE ROI and BF-unlabeled ROI – Error distances between transformed HE landmarks and the original BF ones are then computed

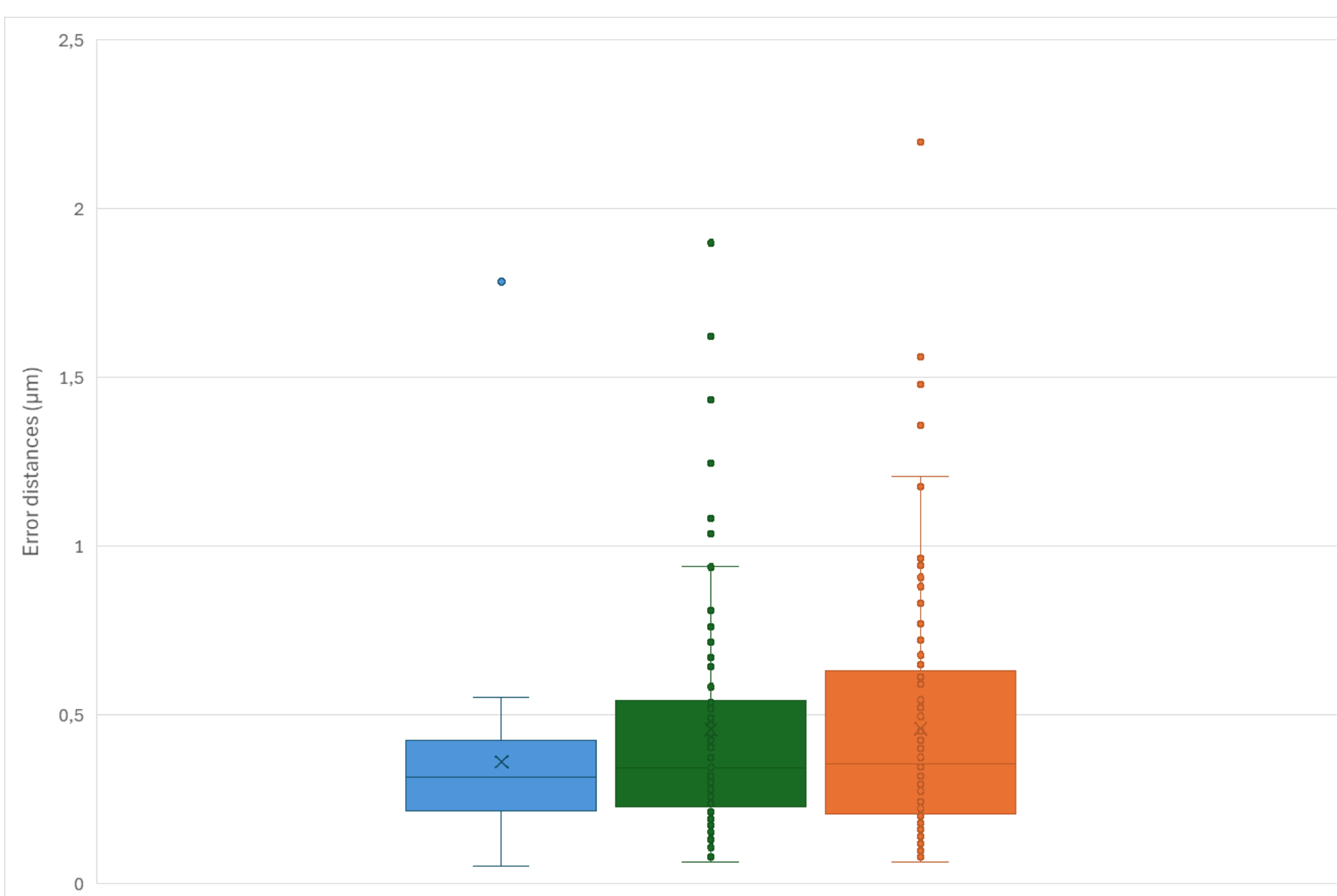

Figure 5. Distance errors of auto-registered landmarks for 3 WSIs from prostate TEST set

5 Deep Learning algorithm – Metrics and training dataset sorting

In digital pathology, deep learning algorithm must be trained with at least pair of patch images : the blank image to be stained and the Ground truth H&E image used specifically for the training. However,

This is a provisional file, not the final typeset article

as the tissue is dehydrated and stained between the first acquisition and the reference acquisition, pixel translation, global morphisms and local tissue morphisms occur. To achieve an efficient training, and remove any bias, the morphism between the images must be compensated and the image must be registered as good as possible. Generally, the registration is done with pixel-size precision, but few large morphisms cannot be fully compensated at this precision.

Structural Similarity Index ($SSIM$) and Multi-Scale SSIM ($MS - SSIM$) are pixel-level metrics used both to evaluate performance and as mathematical thresholds for curating image datasets. They enable the exclusion of poorly registered images, such as those with missing tissue or excessive morphological discrepancies between image to be stained and ground truth image.

As shown in Figure 6, $SSIM$ is highly sensitive to pixel shifts, exhibiting a 40% drop when shifts exceed 2 pixels. In contrast, $MS - SSIM$ demonstrates lower sensitivity, making it a more robust metric for assessing deep learning performance.

Additionally, we employed $SSIM$ to filter out patch images affected by chemical H&E staining artifacts, such as significant morphological distortions or tissue delamination. A threshold of $SSIM < 0.4$, corresponding to shifts exceeding 3 pixels, was applied for exclusion. The filtering methodology was as follows :

- The initial test set consisted of 14,803 patches.
- The $SSIM$ was then computed for this set to identify potentially low-quality cases.
- All patches with $SSIM < 0.4$ were subsequently inspected individually. Only patches exhibiting severe morphological distortions or large spatial shifts, which could not be considered of sufficient quality or accuracy for quantitative evaluation, were excluded.
- Patch images with insufficient tissue content were also excluded. Finally, 13,070 patches remained. We only kept the tissue areas by removing the background to avoid any bias (leading to an overestimation of the metrics).

Importantly, patch images with low $SSIM$ values attributable solely to poor inference were retained for the quantitative evaluation.

In the Figure 7, we summarize the metric values obtained with the Deep Learning model on the patch images used during the test. Notably, an $MS - SSIM$ value of 0.8, achieved by our model, aligns with sub-2-pixel shift equivalence in image quality.

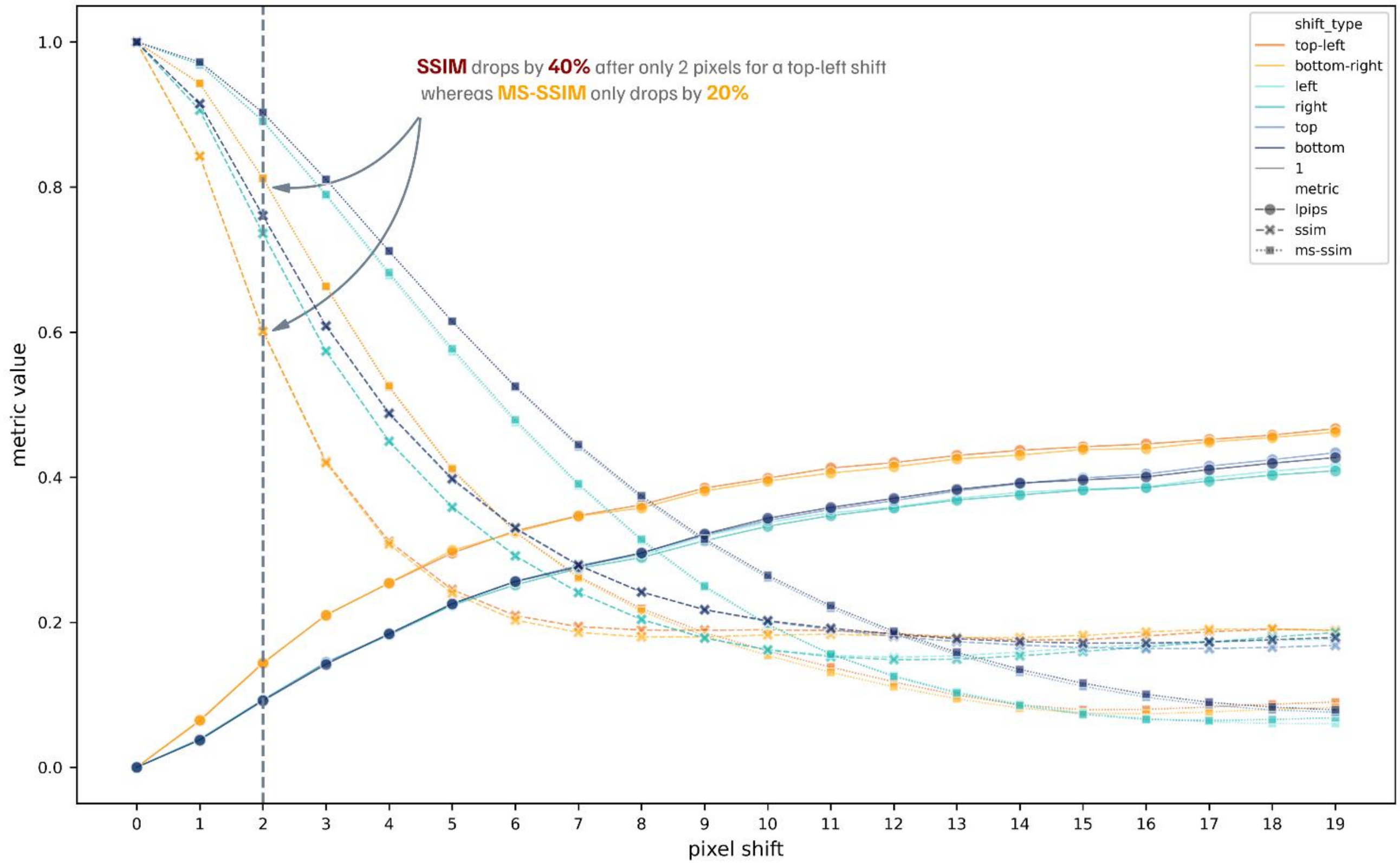

Figure 6. Pixel shift impact on the metrics – Pixel shift between two identical image (0 means no shift, same images)

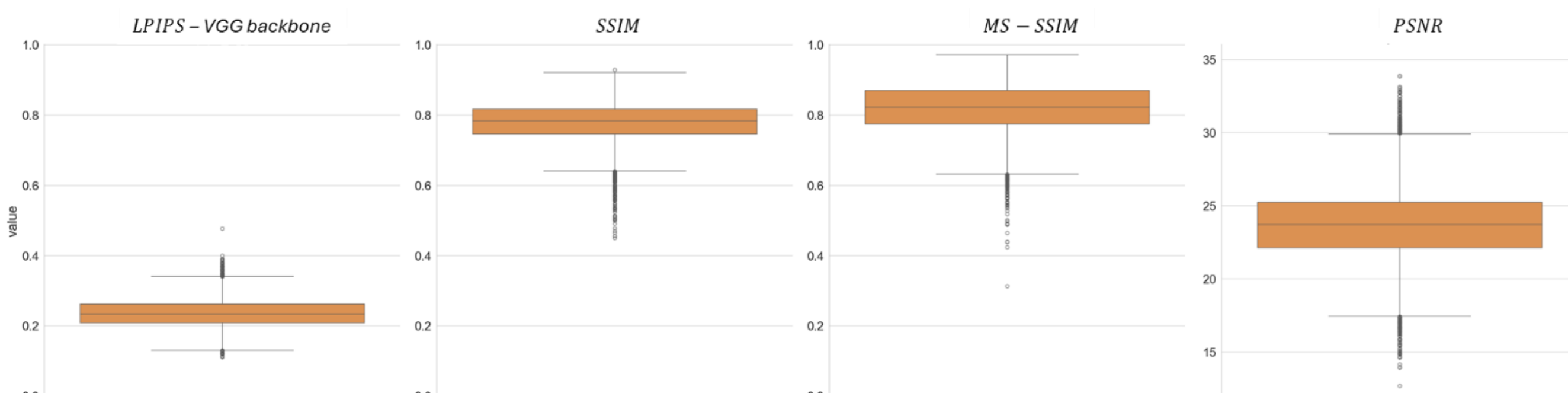

Figure 7. Metrics (averages and deviations)

The low-resolution 16-image IR stack offers localized spectral fingerprints at an 8-µm pixel resolution. A 256×256 pixel BF patch maps directly to a 16×16 pixel region in the IR domain, where each pixel spatially averages the IR fingerprint over 64 µm². This provides 16 wavelengths reflecting the IR

This is a provisional file, not the final typeset article

transmission of individual or multiple cells. We illustrate this mechanism in

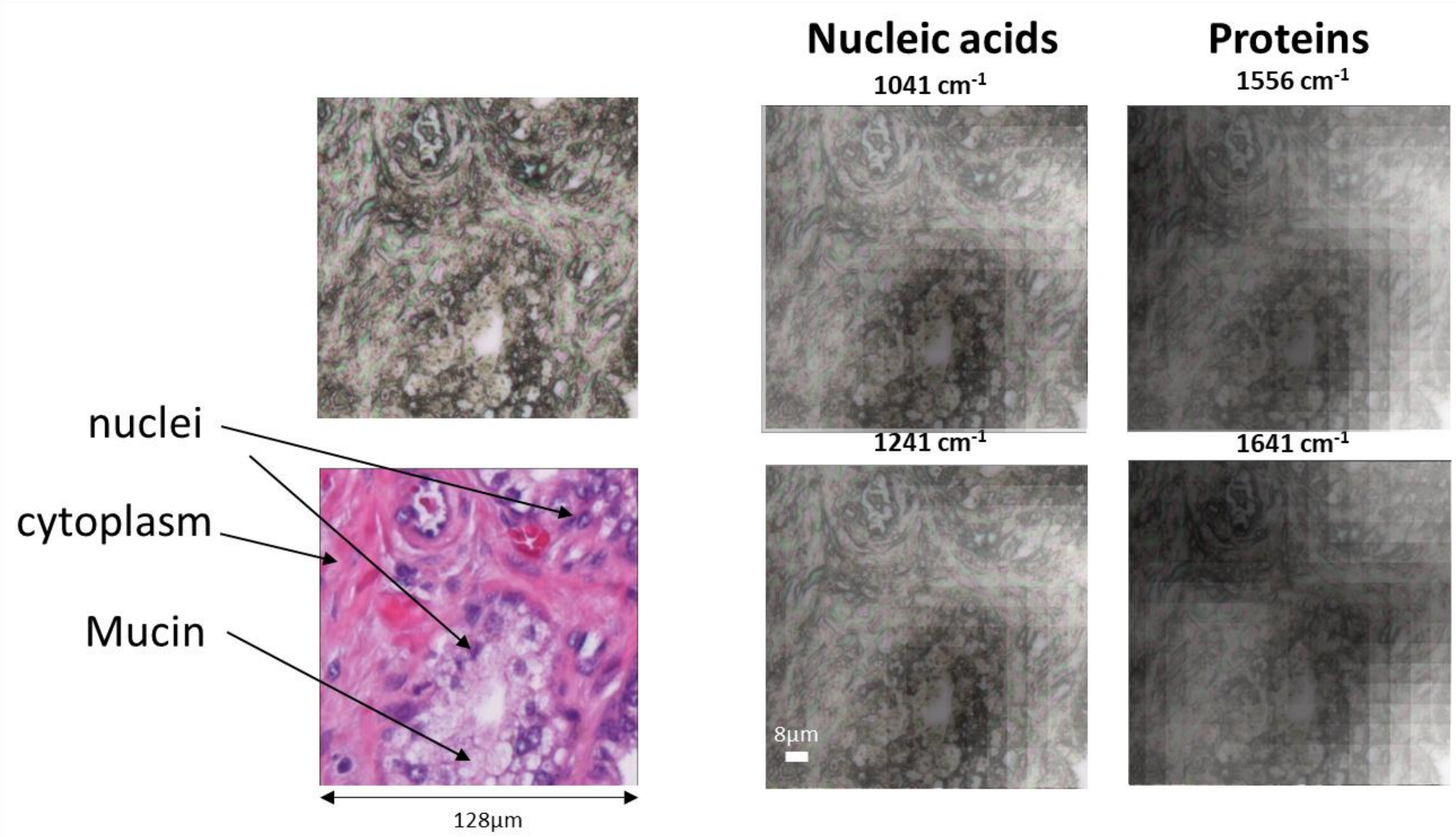

Figure 8, which presents four IR patches superimposed onto the BF patch of an unstained tissue.

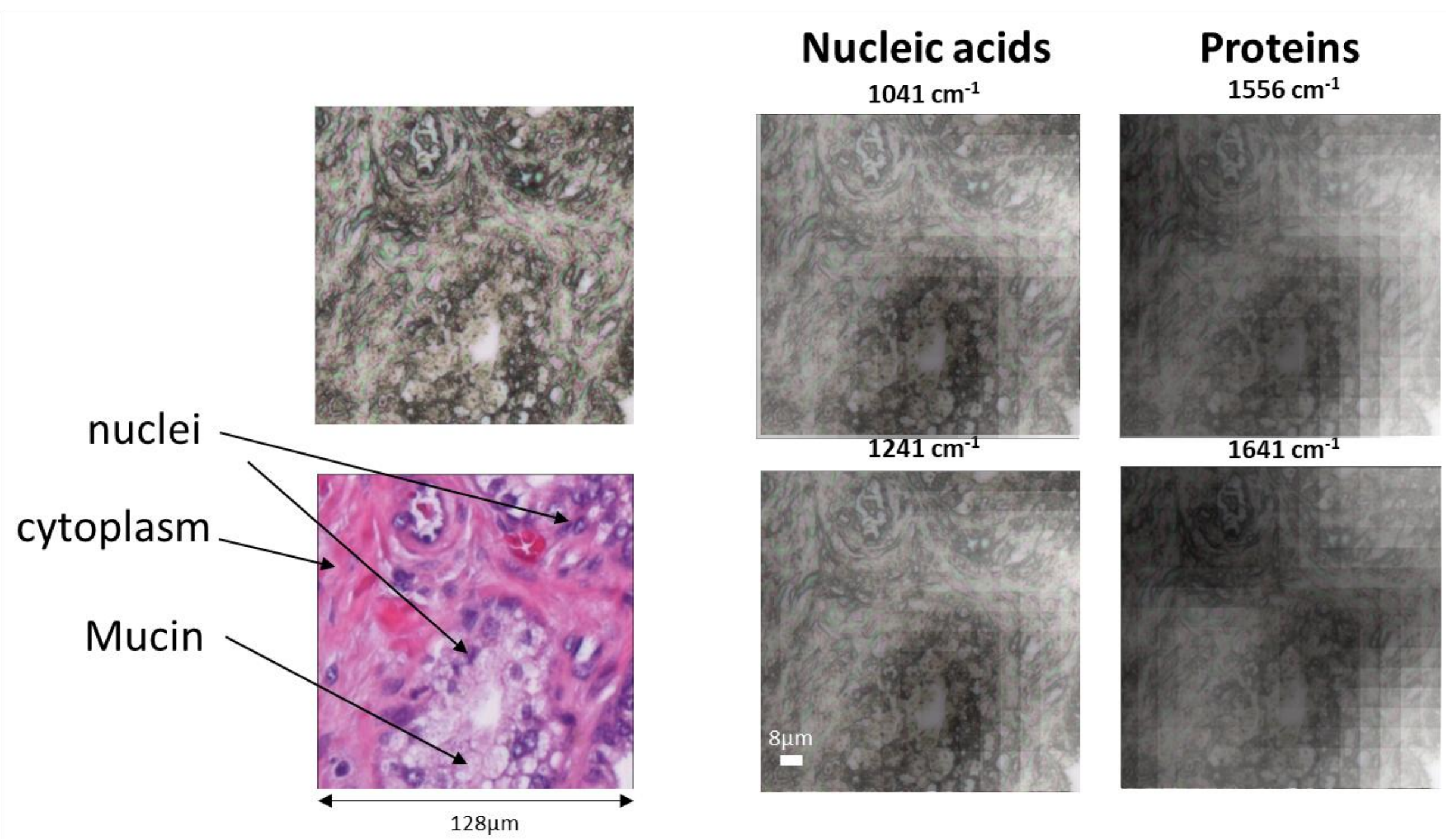

Figure 8. Spatial correlation between IR absorption and tissue morphology. Overlay of IR patches (nucleic acid and protein wavelengths) versus an unstained BF patch. High absorption of nucleic-

acid-targeted IR light highlights primarily nucleus-rich regions, while protein-targeted IR light highlights primarily cytoplasm-rich areas

6 Results – Preclinical evaluation

The 109 ROIs selected to test the digital staining model performance (see Table 1 and Table 2) was also used in a preclinical evaluation. In particular, the evaluation was built to answer to the question :

Is the image quality of the inferences equivalent to the quality exhibited by the ground truth images ?

We considered three mean cellular elements : Nuclear components, morphological characteristics of the cytoplasm, extracellular matrix components (see Table 3 of the main text and Table 5 below).

Rating	Comment
4/4 (Optimal)	Excellent, near perfect
3/4 (Good)	Good, with a few areas that could be improved
2/4 (Bad)	Average quality, but sufficient to establish a diagnosis or prognosis.
1/4 (Unsuitable)	Insufficient, which may result in diagnostic or prognostic errors.

Table 5. Rating criteria for each cellular elements

The scores extracted from the pathologist ratings, presented in the Figure 9, demonstrate that the inferred image quality and ground truth image quality are very close if we consider each pathologist separately. The pathologists are in good agreement regarding the nuclei and cytoplasm but rate differently for collagen. The dendrogram in the Figure 10 confirms that the pathologist rating behaviors are very close for nuclei and cytoplasm. On the contrary, the rating behavior are rather dissimilar for the collagen, one pathologist being more strict regarding the color contrast.
A careful study of the Figure 9 and Figure 10 informs on the expected results for the hypothesis test null-hypothesis H_0, inferences and ground truths are indistinguishable :

- Collagen : even if pathologist ratings are different, the scores by pathologist for inferred ROI and ground truth ROI are very similar.
- Nuclei : The scores of P_2 obtained on inferences and ground truths are similar and we expect that H_0 cannot be rejected. For P_1, H_0 may be rejected.
- Cytoplasm: The conclusion may be the opposite : H_0 rejected for P_2, H_0 cannot be rejected for P_1.

These conclusions are indeed fully verified with the $\chi^2 - test$ presented in the main text.

This is a provisional file, not the final typeset article

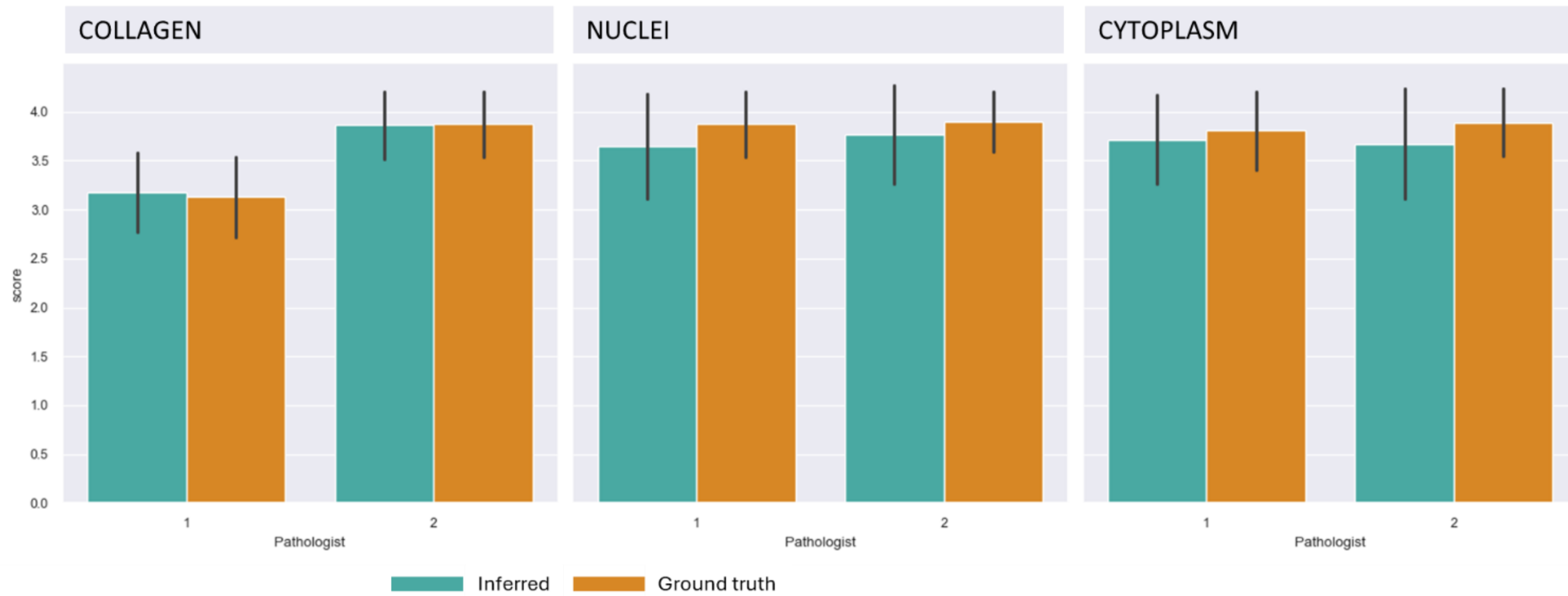

Figure 9. Scores for collagen, nuclei and cytoplasm extracted from pathologist ratings – Green : Inferred – Orange : Real

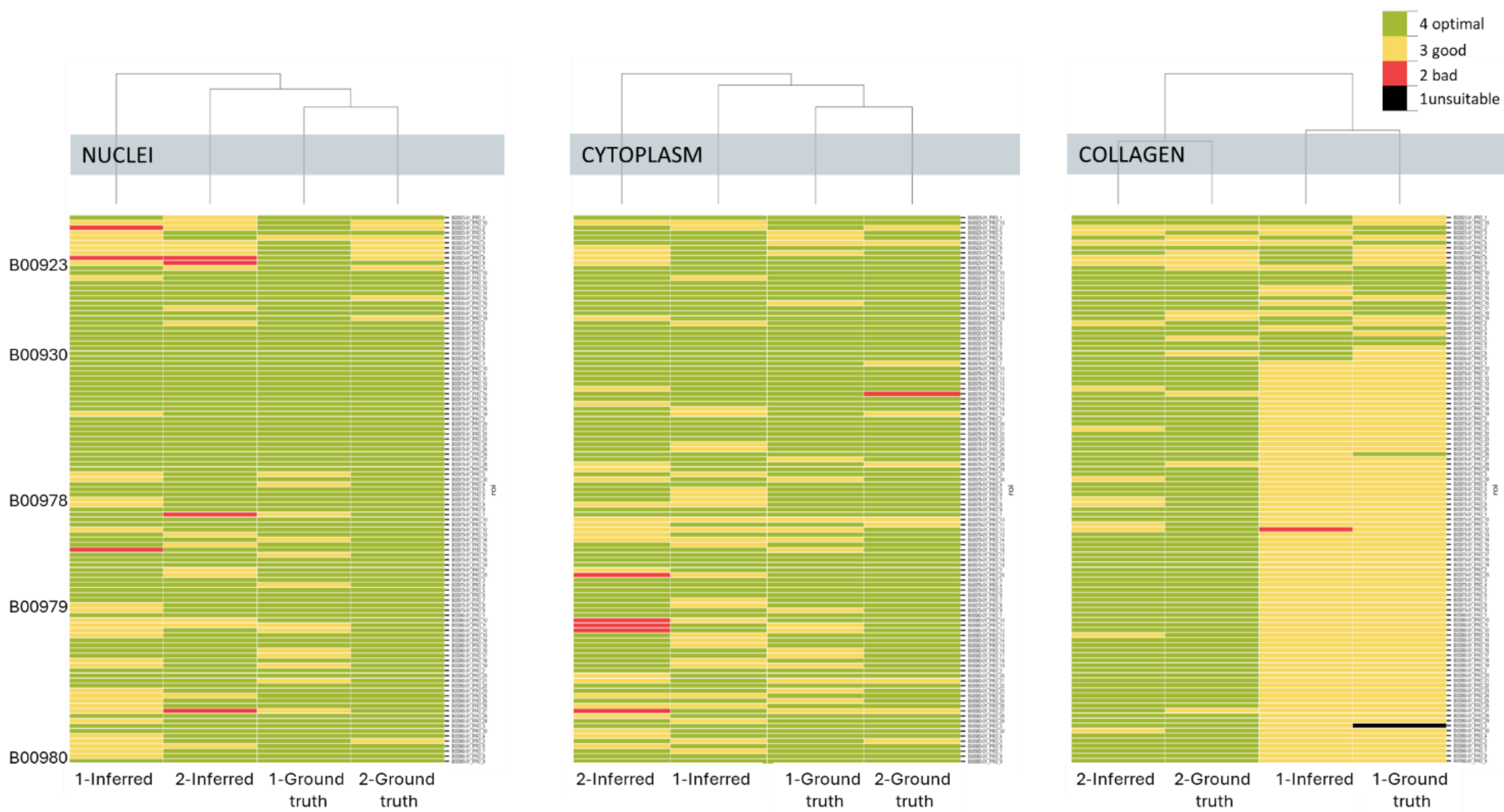

Figure 10. Dendrogram of stain quality scoring
